# A microstructurally motivated framework to study autoregulation in the coronary circulation

**DOI:** 10.1113/JP291222

**Published:** 2026-06-16

**Authors:** Matthew J. Eden, Hamidreza Gharahi, Victoria E. Sturgess, Domingo E. Uceda, Gregory M. Dick, Seungik Baek, Daniel A. Beard, Johnathan D. Tune, C. Alberto Figueroa

**Affiliations:** ^1^ Department of Surgery Section of Vascular Surgery University of Michigan Ann Arbor Michigan USA; ^2^ Department of Biomedical Engineering University of Michigan Ann Arbor Michigan USA; ^3^ Department of Physiology and Anatomy University of North Texas Health Science Center Fort Worth Texas USA; ^4^ Department of Mechanical Engineering Michigan State University East Lansing Michigan USA; ^5^ Department of Molecular and Integrative Physiology University of Michigan Ann Arbor Michigan USA

**Keywords:** cardiovascular regulation, computational modelling, constrained mixture theory, coronary physiology, homeostatic optimization

## Abstract

**Abstract:**

Coronary autoregulation maintains relatively constant myocardial blood flow over a wide range of perfusion pressures through myogenic, shear‐dependent and metabolic control mechanisms. Understanding this phenomenon is challenging due to the coupled nature of these mechanisms and their heterogeneous effects throughout the coronary tree. In this study, we developed a framework to study coronary autoregulation based on constrained mixture theory. The framework simulates autoregulation at three myocardial depths (subepicardium, midwall and subendocardium), calibrated using extensive literature data. Coronary trees at each myocardial depth are constructed via a homeostatic optimization approach to determine morphological and haemodynamic characteristics. Each vessel is endowed with passive and active mechanical properties, governed by a microstructurally motivated wall model. Autoregulatory stimuli from myogenic, metabolic and shear‐dependent mechanisms modulate vascular smooth muscle tone, enabling the framework to reproduce autoregulatory responses, experimentally measured transmural flow ratios and vessel diameter changes with variations in perfusion pressure. The framework also incorporates phasic dynamics by extending Womersley's theory to account for time‐varying intramyocardial pressures, successfully capturing key features of coronary flow waveforms. Sensitivity analysis highlights metabolic mechanisms as primary contributors to autoregulatory function, with the myogenic response playing an important role and shear‐dependent control having minimal contribution. Additionally, the framework demonstrates how changes in vessel microstructure (e.g. collagen stiffening or impaired smooth muscle contractility) affect autoregulatory capacity, providing mechanistic insight into pathophysiological states. This microstructurally motivated framework offers a novel approach for hypothesis testing in coronary autoregulation while providing a unified platform for describing processes spanning short‐term tone regulation and long‐term vascular remodelling.

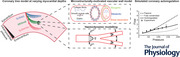

**Key points:**

- Coronary autoregulation is defined as the capability of the coronary circulation to maintain the blood supply to the heart over a range of perfusion pressures. This phenomenon is facilitated through intrinsic mechanisms that control the vascular resistance by regulating the function of smooth muscle cells.
- This paper presents a microstructurally motivated coronary autoregulation framework that uses a non‐linear continuum mechanics approach to account for the morphometry and vessel wall composition in three coronary trees in the subepicardial, midwall and subendocardial layers of the myocardium.
- The model is calibrated against diverse experimental data from the literature and is used to study heterogeneous autoregulatory response in the coronary trees.
- This model drastically differs from previous models and is suited to the study of long‐term pathophysiological growth and remodelling phenomena in coronary vessels.

## Graphical abstract

**Abstract figure legend** A microstructurally motivated framework was constructed to simulate coronary autoregulation in symmetrically bifurcating trees at three myocardial depths: subepicardial (subepi), midwall and subendocardial (subendo). Wall mechanics of individual vessels are governed by a constrained‐mixture model, incorporating elastin, collagen fibres, and vascular smooth muscle cells. The tone of smooth muscle cells is modulated by myogenic, metabolic and shear‐dependent control signals, producing changes in resistance and regulating flow. Haemodynamics are computed throughout the coronary trees, accounting for transmurally varying intramyocardial pressure. The calibrated framework reproduces key features of coronary pressure–flow autoregulation.

## Introduction

The myocardium extracts approximately 70–80% of oxygen from coronary arterial blood under resting conditions. Therefore, shifts in metabolic demand (myocardial oxygen consumption, $\mathrm{MV}O_{2}$) are largely met by changes in coronary blood flow (Feigl, 1983). Coronary flow regulation is the result of vessel diameter modulation via local control of the active stress in the vascular smooth muscle cells (SMCs) (Goodwill et al., 2017). One aspect of coronary flow regulation is referred to as pressure‐flow autoregulation, in which changes in coronary perfusion pressure are met with adjustments in microvascular resistance to maintain a relatively constant coronary blood flow at a given $\mathrm{MV}O_{2}$.

Coronary autoregulation relies on SMC reactivity, regulated through myogenic, metabolic and shear‐dependent control mechanisms. Myogenic control is the intrinsic response of SMCs to changes in local wall hoop stress (Kuo et al., 1990). Metabolic control is a local feedback mechanism where either an increase in local oxygen consumption or oxygen extraction from the myocardial blood supply triggers a vasodilatory signal (Feigl, 1983). Shear‐dependent control is a dilatory mechanism mediated via shear‐induced production of nitric oxide (NO) by the endothelial cells. Studies have shown that the relative strengths of these control mechanisms vary throughout the coronary circulation (Tiefenbacher & Chilian, 1998). Myogenic control has been observed to be small in precapillary arterioles and large in 50–150 µm diameter arteries (Liao & Kuo, 1997). Shear‐dependent control is most active in the arteries and large arterioles (Kuo et al., 1995). Lastly, the signal for the metabolic feedback mechanism is initiated in the capillaries, conducted upstream, and gradually decays for vessels >150 µm in diameter (Kanatsuka et al., 1989).

In addition to the variable strengths of the autoregulatory mechanisms, the coronary circulation contains several other important heterogeneities. Non‐uniform myocardial‐vessel interactions result in subendocardial vessels experiencing a greater intramyocardial pressure than those in the subepicardium (Zhang et al., 2008). Furthermore, vessel morphometry varies transmurally across the myocardium, with vessels of the same diameter reported to be thicker in the subepicardium *versus* the subendocardium (Choy & Kassab, 2009). Collectively, anatomical and functional differences across the myocardium result in a transmural variation in coronary autoregulation (Boatwright et al., 1980). The inherent heterogeneous nature of the coronary circulation, combined with the challenges associated with obtaining *in vivo* measurements, has made our understanding of coronary autoregulation limited and mostly phenomenological. Developing computational models that integrate available experimental data on morphometry, biomechanical and physiological responses can enhance our understanding of coronary autoregulation in health, disease and treatment planning conditions.

Coronary autoregulation has been the subject of numerous modelling studies over the last 50–60 years. Virtually all such studies have relied on lumped‐parameter (0D) approaches. Liao and Kuo (1997) and Cornelissen et al. (2002) investigated the interaction and balance between autoregulatory mechanisms in the coronary circulation. While these studies incorporated the heterogeneity of the microvascular response, they partitioned the coronary tree into four compartments and ignored the interactions between vessels and myocardium. Pradhan et al. (2016) developed a data‐driven closed loop model of regulation using *in vivo* data on coronary flow and oxygen extraction in response to exercise‐induced changes in demand and perfusion pressure. The Pradhan model, which represents parallel control pathways using a block‐diagram approach, does not represent explicit spatial features. Namani et al. (2018) developed a coronary microcirculation model that integrated dynamic effects of flow with myogenic, shear‐dependent and metabolic feedback control mechanisms in subendocardial and subepicardial vessels. Their model considered simple pressure–diameter rules to define the vessel behaviour. Recently, Gharahi et al. (2022) and Sturgess et al. (2024) utilized a non‐linear three‐layer lumped parameter network to investigate autoregulation across the myocardium under varying metabolic demands. While all these studies provide reasonable descriptions of autoregulatory responses, their inherent 0D nature complicates their interpretation. 0D models make it difficult to incorporate microstructurally derived information on structure and function on image‐based networks of vessels. Lastly, given that the short‐term regulation of SMC tone and the long‐term vascular growth and remodelling processes, including pathophysiological responses, are highly intertwined (van den Akker et al., 2010), we submit that there is a pressing need to develop microstructurally motivated models of coronary autoregulation.

Constrained mixture models (CMMs) have been widely applied to describe the non‐linear mechanical behaviour of arterial tissue, accounting for the contributions of the main load‐bearing constituents (e.g. collagen, elastin and SMCs) (Bellini et al., 2014; Ferruzzi et al., 2010; Reesink & Spronck, 2019). This theory provides a formal means to represent mechanical function of a vessel based on the properties and relative mass fractions of its constituents. Using this theory, long‐term growth and remodelling is represented by changes in the composition of the vessel wall. Previous applications of CMMs in vascular mechanics have focused on large arteries where homeostatic stress has been assumed constant. However, wall shear stress and hoop stress have been found to be size‐dependent through the vasculature (Guo & Kassab, 2004; Pries et al., 2005; Stepp et al., 1999). More recently, CMMs have been applied to study flow‐mediated growth and remodelling (fluid–solid growth) in both large vessels (Baek et al., 2007; Figueroa et al., 2009) as well as networks of vessels (Gharahi et al., 2023; Szafron et al., 2023). However, CMMs have yet to be used to study autoregulation in vascular networks.

The goal of this study is to develop a microstructurally motivated computational framework of coronary autoregulation, utilizing a recent homeostatic optimization model based on a CMM developed by Gharahi et al. (2023). The framework provides a means to estimate baseline activation of SMCs and homeostatic stresses, while incorporating experimental data on pressure–diameter, tree morphometry and pulsatile haemodynamics. The utility of the proposed microstructurally motivated framework is demonstrated through different examples of short‐term adaptations to changes in perfusion pressure. A key benefit of this framework is that it provides a method to probe relative contributions of the different autoregulatory mechanisms across different vessel sizes and myocardial depths.

## Methods

### Framework overview and calibration strategy

**Figure 1:**
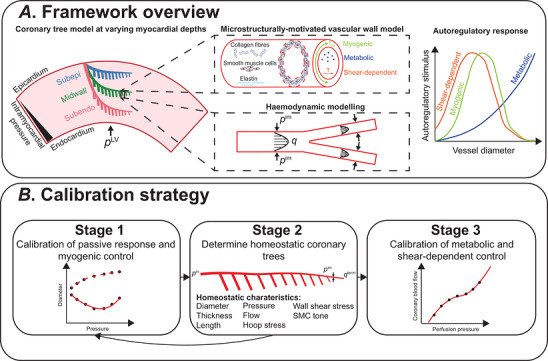
Microstructurally motivated framework of coronary autoregulation and calibration strategy Overview of the coronary tree autoregulation framework (*A*), consisting of subtrees at three myocardial depths: subepi, midwall and subendo subjected to spatially varying intramyocardial pressure ($p^{im}$). Vessel mechanics are governed by a microstructure‐motivated wall model incorporating collagen, SMCs and elastin. SMC activation is controlled by myogenic, metabolic and shear‐dependent autoregulatory stimuli, which are calculated through haemodynamic modelling. The model undergoes a three‐stage calibration procedure (*B*) to establish passive and active properties that reproduce coronary pressure–flow autoregulation.

Figure 1*A* shows an overview of the framework. Anatomically, we considered three different symmetrically bifurcating coronary trees at three different myocardial depths: subepicardium (subepi), midwall and subendocardium (subendo), which are subjected to an intramyocardial pressure. Each subtree consists of 12 vessel generations. Vessel wall mechanics are governed by a microstructurally motivated model based on a CMM formulation, treating collagen, SMCs and elastin as load‐bearing constituents. SMC activation modulates vessel diameter, and is controlled by myogenic, metabolic and shear‐dependent autoregulatory stimuli and determined through haemodynamic modelling. Our framework is calibrated using extensive literature data and considers the relative contributions of the three autoregulatory stimuli.

Framework calibration entails a three‐stage procedure (Fig. 1*B*). In Stage 1, passive and myogenic vessel response is calibrated using pressure–diameter myography data (Liao & Kuo, 1997). In Stage 2, the Gharahi et al. (2023) model is used to define homeostatic morphometry and haemodynamics of the three coronary subtrees. An iterative process between Stages 1 and 2 is performed until the vessel mechanics and homeostatic quantities (haemodynamics, morphometry and SMC tone) converge. Lastly, in Stage 3, metabolic and shear‐dependent autoregulatory responses are calibrated to match a given coronary pressure–flow relationship (Dick et al., 2018). The result of this calibration procedure is a tree model endowed with morphological, haemodynamic, structural and autoregulatory characteristics of the coronary circulation.

### Microstructurally motivated vessel wall model

The microstructurally motivated wall model, which governs the passive and active behaviour for all vessels, is presented here, as well as the model of SMC activation and autoregulatory stimuli.

#### CMM formulation

Vessel wall mechanics are described with a CMM formulation, considering the main load‐bearing constituents: elastin, collagen and SMCs (Humphrey & Rajagopal, 2002). A strain energy function w is used to describe the relationship between stress and strain tensors in a non‐linear manner for each constituent. CMMs incorporate the microstructural properties such that wall constituents are constrained to deform together but have distinct mechanical properties and stresses (Humphrey, 2002). Each constituent i has a strain energy density ($w^{i}$), which is a function of deformation gradient that maps from constituent i ’s stress‐free configuration to the deformed configuration. The total strain energy density for a vessel is the sum of its constituents: $w=\sum_{i}w^{i}$. The function consists of passive contributions for elastin ($w_{pass}^{e})$ and collagen ($w_{pass}^{c})$, as well both passive ($w_{pass}^{SMC})$ and active ($w_{act}^{SMC})$ contributions for SMC. Therefore, we have:

(1) $$w=w_{pass}^{e}+w_{pass}^{c}+w_{pass}^{SMC}+w_{act}^{SMC}.$$

A neo‐Hookean model is used for elastin (treated as an isotropic constituent with coefficient $c_{1}$), and a Holzapfel exponential model is used for the collagen fibres (with coefficients $c_{2}$ and $c_{3}$) and the SMCs’ passive response (with coefficients $c_{4}$ and $c_{5}$).

Critically for this work, the active SMC response is modelled as:

(2) $$w_{act}^{SMC}=A\frac{S_{max}M_{R}^{SMC}}{\rho_{wall}}\left(\lambda_{\theta }+\frac{{\left(\lambda_{M}-\lambda_{\theta }\right)}^{3}}{3{\left(\lambda_{M}-\lambda_{0}\right)}^{2}}\right),$$

where A is an SMC activation level (0<A<1), $S_{max}$ is the maximum active stress, $M_{R}^{SMC}$ is the mass per unit area of SMCs, $\rho_{wall}$ is the density of arterial tissue, $\lambda_{\theta }$ is the vessel circumferential stretch, and $\lambda_{0}$ and $\lambda_{M}$ are the zero and maximum active tension stretches, respectively. Other important material parameters in CMMs of vascular tissue are: (1) homeostatic pre‐stretches at which each constituent is deposited ($G_{\theta }^{e}$, $G_{z}^{e}$, $G_{h}^{c}$ and $G_{h}^{SMC}$), and (2) the fraction of the vessel wall occupied by each constituent (mass fractions $\nu^{e}$, $\nu^{c}$ and $\nu^{m}$). Details on elastin, collagen and SMC constituent orientations within the vessel wall, as well as other mathematical forms of the CMMs are summarized in Appendix A and available in greater detail elsewhere (Baek et al., 2007).

Using membrane theory (Humphrey, 2002), the circumferential wall tension ($T_{\theta \theta }$) of a thin‐walled vessel is calculated from its strain energy density as:

(3) $$T_{\theta \theta }=\frac{1}{\lambda_{z}}\;\frac{\partial w}{\partial \lambda_{\theta }},$$

where $\lambda_{z}$ is the axial stretch of the vessel. Furthermore, mechanical equilibrium is given by Laplace's law:

(4) $$T_{\theta \theta }=\frac{p^{tv}D}{2}\;,$$

where $p^{tv}$ is transvascular pressure and D is lumen diameter. Note, $p^{tv}$ is the difference between lumen pressure (p) and the intramyocardial pressure ($p^{im}$): $p^{tv}=p-\;p^{im}$. By combining Equations (3) and (4), the vessel diameter can be determined. Autoregulation is incorporated within the CMM formulation by adjusting the SMC activation A in response to autoregulatory stimuli.

#### Modelling of autoregulatory stimulus

SMC activation is modulated between fully dilated (A=0) and maximally constricted (A=1). Following Carlson et al. (2008), A is assumed to be sigmoidal and to depend on the total SMC stimulus ($s_{total}$):

(5) $$A=\frac{1}{1+e^{-s_{total}}}\;,$$

where $s_{total}$ depends on myogenic ($s_{myo}$), metabolic ($s_{meta}$) and shear‐dependent ($s_{\tau }$) stimuli. Furthermore, SMCs maintain a basal tone under homeostatic conditions ($s_{h}$) (Martinez‐Lemus et al., 2003). Thus, $s_{total}$ can be expressed as:

(6) $$s_{total}=s_{myo}+s_{meta}+s_{\tau }+s_{h}.$$

It is important to point out that under the homeostatic state $s_{h}$ contains contributions from each autoregulatory mechanism, even though at the homeostatic state the three different signals (myogenic, metabolic and shear‐dependent) are zero by construction (see below). Since the relative strength of these control mechanisms depends on vessel size (Arciero et al., 2008; Goodwill et al., 2017; Liao & Kuo, 1997; Stepp et al., 1999), normalized diameter‐dependent response curves (0≤ϕ≤1) for the myogenic ($\phi_{myo}\left(D\right)$), metabolic ($\phi_{meta}\left(D\right)$) and shear‐dependent ($\phi_{\tau }\left(D\right)$) control mechanisms are defined below.

##### Myogenic control

The myogenic stimulus $s_{myo}$ is assumed to be linearly dependent on wall tension $T_{\theta \theta }$ (Carlson et al., 2008):

(7) $$s_{myo}=\phi_{myo}\;a_{myo}\left(\frac{T_{\theta \theta }-\;T_{\theta \theta_{h}}}{T_{\theta \theta_{h}}}\right),$$

where $a_{myo}$ is a myogenic scaling coefficient and $T_{\theta \theta_{h}}$ is the homeostatic wall tension. From Stage 1 model calibration, we obtain $a_{myo}$ and $\phi_{myo}$ at four representative vessel sizes (D = 180, 95, 62 and 37 µm), as discussed in Appendix B, using the data of Liao & Kuo (1997). To extrapolate $\phi_{myo}$ to other vessel diameters, we assume no myogenic response (i.e. $\phi_{myo}=0$) for the precapillary arterioles and for D >500 µm (Dick et al., 2018). $\phi_{myo}$ is interpolated across all vessel diameters using a power‐law scheme. As discussed in Appendix B, each of the three subtrees has distinct $\phi_{myo}$ distributions, denoted $\phi_{myo}^{subepi}$, $\phi_{myo}^{midwall}$ and $\phi_{myo}^{subendo}$.

##### Metabolic control

Changes in $\mathrm{MV}O_{2}$ are assumed to be matched by proportional changes in flow to meet the oxygen demand (Arthurs et al., 2016). Based on this assumption, the metabolic stimulus $s_{meta}$ is linearly dependent on the terminal arteriole flow rate, regardless of the underlying physiological mechanisms involved (Namani et al., 2018):

(8) $$s_{meta}=\phi_{meta}a_{meta}\;\left(\frac{q^{term}-\hat{q}\left(MVO_{2}\right)}{\hat{q}\left(MVO_{2}\right)}\right),$$

where $a_{meta}$ is a metabolic scaling coefficient, $\hat{q}\left(\mathrm{MV}O_{2}\right)$ is the target flow rate determined by a baseline metabolic demand and $q^{term}$ is the terminal arterioles flow rate. The metabolic response curve $\phi_{meta}$ assumes that metabolic signals originate in the capillaries and propagate upstream via endothelial gap junctions (Namani et al., 2018):

(9) $$\phi_{meta}=e^{-\frac{L_{p}\left(D\right)}{L_{0}}}\;,$$

where $L_{p}\left(D\right)$ is the path length from the midpoint of a vessel with diameter D to the capillaries, and $L_{0}$ is a characteristic decay length, assumed to be $L_{0}$ = 0.001 m (Namani et al., 2018).

##### Shear‐dependent control

An increase in wall shear stress τ induces relaxation of SMCs. The shear‐dependent stimulus ($s_{\tau }$) is modelled as:

(10) $$s_{\tau }=-\phi_{\tau }a_{\tau }\left(\frac{\tau -\;\tau_{h}}{\tau_{h}}\right),$$

where $a_{\tau }$ is a shear‐dependent scaling coefficient and $\tau_{h}$ is a homeostatic wall shear stress. The negative sign indicates a vasodilatory response from an increase in wall shear stress. Measurements of fractional dilatation induced by shear stress in swine coronary arteries at four vessel sizes (D = 180, 95, 62 and 37 µm) from Liao and Kuo (1997) were used as a proxy for the shear‐dependent normalized response $\phi_{\tau }$. Similar to myogenic control, $\phi_{\tau }$ is extrapolated to the entire coronary tree assuming negligible shear‐dependent control (i.e. $\phi_{\tau }=0$) for the precapillary arterioles and for vessel dimeters ≥730 µm (Namani et al., 2018). A power‐law scheme is used to interpolate $\phi_{\tau }$ across all vessel diameters.

##### Homeostatic stimulus

Note that at the homeostatic state $A=A_{h}$ and $s_{total}=s_{h}$. The homeostatic SMC stimulus $s_{h}$ and $A_{h}$ are related by:

(11) $$s_{h}=-\ln\left(\frac{1-A_{h}}{A_{h}}\right).$$

**Figure 2:**
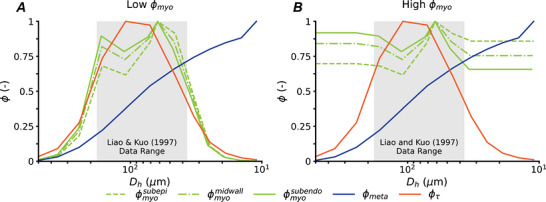
Normalized myogenic response profiles *A*, Low $\phi_{myo}$ and *B*, High $\phi_{myo}$ profiles for myogenic response curves. Metabolic ($\phi_{meta}$) and shear‐dependent ($\phi_{\tau }$) response curves are identical in both examples. The grey box indicates vessel diameters in the Liao and Kuo (1997) dataset.

Two examples of normalized diameter‐dependent response curves are presented in Fig. 2. Once the myogenic responses are calibrated for vessels within the Liao & Kuo (1997) dataset (180 > *D* > 37 µm, see shaded region), two different assumptions for myogenic responses outside this range are considered. In Fig. 2*A* (Low $\phi_{myo}$), the myogenic responses are assumed to drop to zero, whereas in Fig. 2*B* (High $\phi_{myo}$), the myogenic responses are assumed to remain constant outside the range. Note that the High $\phi_{myo}$ profile is not intended to be physiologically realistic, but is used to assess how different response curves may impact modelling results. Results are presented using the Low $\phi_{myo}$ profile, unless stated otherwise.

### A model for intramyocardial pressures

**Figure 3:**
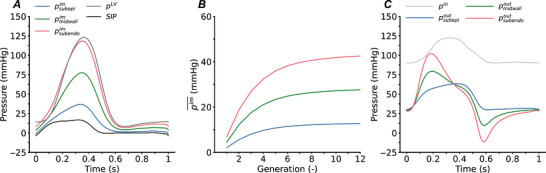
Imposed pressure waveforms on the coronary tree *A*, pulsatile intramyocardial pressures ($p^{im}$) at a normalized myocardial depth of $\beta_{j}\left(D\right)=\beta_{cap,\;j}$ for each coronary subtree, calculated from left ventricular ($p^{LV}$) and shortening‐induced pressure (SIP) via Equation (12). *B*, distribution of mean intramyocardial pressure ($\bar{p^{im}}$) across vessel generations for each coronary subtree, based on the normalized myocardial depth distribution from Equation (13). *C*, inlet and outlet lumen boundary pressure waveforms from Namani et al. (2018).

Intramyocardial pressure ($p^{im}$) depends on a shortening‐induced pressure (SIP) and a cavity‐induced extracellular pressure (CEP) (Algranati et al., 2010). SIP accounts for myofibril contraction during myocyte activation and is assumed to remain constant across the myocardium. CEP is the pressure transmitted from the left ventricle ($p^{LV}$), and is greater in the endocardium than in the epicardium (Arts & Reneman, 1985). The distribution of $p^{im}$ along each subtree j (subepi, midwall, subendo) is modelled as (Sturgess et al., 2024):

(12) $$p_{j}^{im}\left(D\right)=SIP+CEP=SIP+\beta_{j}\left(D\right)\cdot p^{LV},$$

where $\beta_{j}\left(D\right)$ is a normalized myocardial depth distribution, with $\beta_{j}=1$ at the endocardial surface and $\beta_{j}=0$ at the epicardium surface. SIP and $p^{LV}$ waveforms are taken from Namani et al. (2018) (Fig. 3*A*). We assign $\beta_{j}\left(D\right)$ distributions that anatomically represent coronary trees penetrating the myocardium from the epicardial surface, and approaching capillary beds located at normalized myocardial depths $\beta_{cap,\;j}$ of 1/6, 1/2 and 5/6 for the subepi, midwall and subendo layers, respectively:

(13) $$\beta_{j}\left(D\right)=\beta_{cap,\;j}\;\frac{L_{p}\left(D\right)}{L_{p_{total}}},$$

where $L_{p}\left(D\right)$ is the path length between the midpoint of a vessel with diameter D to the terminal vessel and $L_{p_{total}}$ is the total subtree path length. The pulsatile $p^{im}$ at the capillary depth $\beta_{j}\left(D\right)=\beta_{cap,\;j}$ is shown in Fig. 3*A* for each coronary subtree, while the distribution of mean intramyocardial pressure ($\bar{p^{im}}$) across subtree generations is presented in Fig. 3*B*.

### Haemodynamic modelling

Vessel fluid–solid interaction is modelled using Womersley's solution for oscillatory flow in deformable tubes (Womersley, 1957). This approach solves for flow and pressure under linearized continuum assumptions, as described in detail elsewhere (Filonova et al., 2020; Gharahi et al., 2023). The continuum assumption allows pressures and flows to be rapidly computed throughout the coronary tree, circumventing the need to resolve the complex physics (Forouzandehmehr & Shamloo, 2018). In this work, Womersley's solution is extended to account for a time‐ and spatially varying $p^{im}$. Furthermore, a system of equations is constructed to solve haemodynamics throughout the coronary tree.

Given the small diameters of the vessel trees in this study, the interactions between red blood cells and plasma become complex leading to highly non‐linear viscosity (μ) behaviour, for example Fåhræus–Lindqvist effects (Fåhræus & Lindqvist, 1931). To account for some of this complexity while maintaining a continuum approach for solving flow, in this work we have adopted the *in vivo* viscosity law from Pries et al. (1994):

(14) $$\begin{array}{ccc} C & = & \left(0.8+e^{-0.07D}\right)\;\left(-1+\frac{1}{\left(1+10^{-11}D^{12}\right)}\right)\\ & & +\;\frac{1}{\left(1+10^{-11}D^{12}\right)}, \end{array}$$

(15) $$h_{f}=\frac{{\left(1-H_{D}\right)}^{C}-1}{{\left(1-0.45\right)}^{C}-1}\;,$$

(16) $$\mu_{0.45}=6e^{\left(-0.085D\right)}+3.2-2.44e^{-0.06D^{0.645}},$$

(17) $$\mu =\mu_{0}\;\left(1+h_{f}\left(\mu_{0.45}-1\right){\left(\frac{D}{D-1.1}\right)}^{2}\right){\left(\frac{D}{D-1.1}\right)}^{2},$$

where *D* is the vessel diameter, ($H_{D}$) is the haematocrit level and $\mu_{0}$ the blood plasma viscosity taken to be 0.001 Pa.s.

#### Extension of Womersley's solution for time‐varying external pressure

Intramyocardial pressure is assumed to be a known periodic quantity, uniform along a single vessel of length L (i.e. $\frac{\partial p^{im}}{\partial z}=0$). In the frequency domain, flows (q) and transvascular pressures ($p^{tv}$) are related through an admittance matrix (Y) (Mackenzie, 2021):

(18) $$q=Y\;p^{tv}=Y\left(p-\;p^{im}\right),$$

where $q\;={\left[q_{1}\;q_{2}\right]}^{T}$ and $p^{\mathrm{tv}}={\left[p_{1}^{tv}\;p_{2}^{tv}\right]}^{T}$, and subscripts 1 and 2 designate the vessel inlet (z=0) and outlet (z=L), respectively. Details on constructing Y are presented in Appendix C. For non‐zero frequencies (ω≠0), Y is:

(19) $$Y\;=\frac{\frac{\pi D^{2}}{4c\rho_{blood}}\left(1-g\right)}{1-{\left(cos\left(-\frac{\omega L}{c}\right)+isin\left(-\frac{\omega L}{c}\right)\right)}^{2}}\left[\begin{array}{cc} 1+{\left(cos\left(-\frac{\omega L}{c}\right)+isin\left(-\frac{\omega L}{c}\right)\right)}^{2} & -2\left(cos\left(-\frac{\omega L}{c}\right)+isin\left(-\frac{\omega L}{c}\right)\right)\\ -2\left(cos\left(-\frac{\omega L}{c}\right)+isin\left(-\frac{\omega L}{c}\right)\right) & 1+{\left(cos\left(-\frac{\omega L}{c}\right)+isin\left(-\frac{\omega L}{c}\right)\right)}^{2} \end{array}\right],$$

where c is the pulse wave velocity, $\rho_{blood}$ is the blood density, $i=\sqrt{-1}$ and $g=2J_{1}\left(\wedge \right)/\wedge J_{0}\left(\wedge \right)$. $J_{0}$ and $J_{1}$ are Bessel functions of the first kind and $\wedge =i^{\frac{3}{2}}\;\alpha_{Wom}$, where $\alpha_{Wom}=\frac{D}{2}\;\sqrt{\omega \rho_{blood}/\mu }$ is the Womersley number and μ the blood viscosity. For the steady component of Womersley's solution (i.e. ω=0), Y is (Qureshi et al., 2014):

(20) $$Y\;=\frac{\pi D^{4}}{128\mu L}\left[\begin{array}{cc} 1 & -1\\ -1 & 1 \end{array}\right].$$

Thus, Equations (18)–(20) calculate flow and pressure and the ends of a deformable vessel.

#### Womersley's solution for an arterial network

At any bifurcation, conservation of flow and continuity of pressure states:

(21) $$p_{m}\;\left(z=L\right)=p_{d1}\;\left(z=0\right)=p_{d2}\left(z=0\right),$$

(22) $$q_{m}\;\left(z=L\right)=q_{d1}\left(z=0\right)+q_{d2}\left(z=0\right),$$

where m and d1, d2 denote mother and two daughter branches, respectively. By applying Equation (18) to each vessel and Equations (21) and (22) at each bifurcation, we construct a linear system of equations: Cx=b, where C is a coefficient matrix, x the vector of unknown flows and pressures, and b is a vector containing the prescribed boundary conditions and $p^{im}$ waveforms. In this paper, pulsatile haemodynamics are only considered at the homeostatic state, which uses the inlet and outlet pressure boundary conditions taken from Namani et al. (2018) (Fig. 3*C*).

### Framework algorithm: how to calculate an autoregulated state

**Figure 4:**
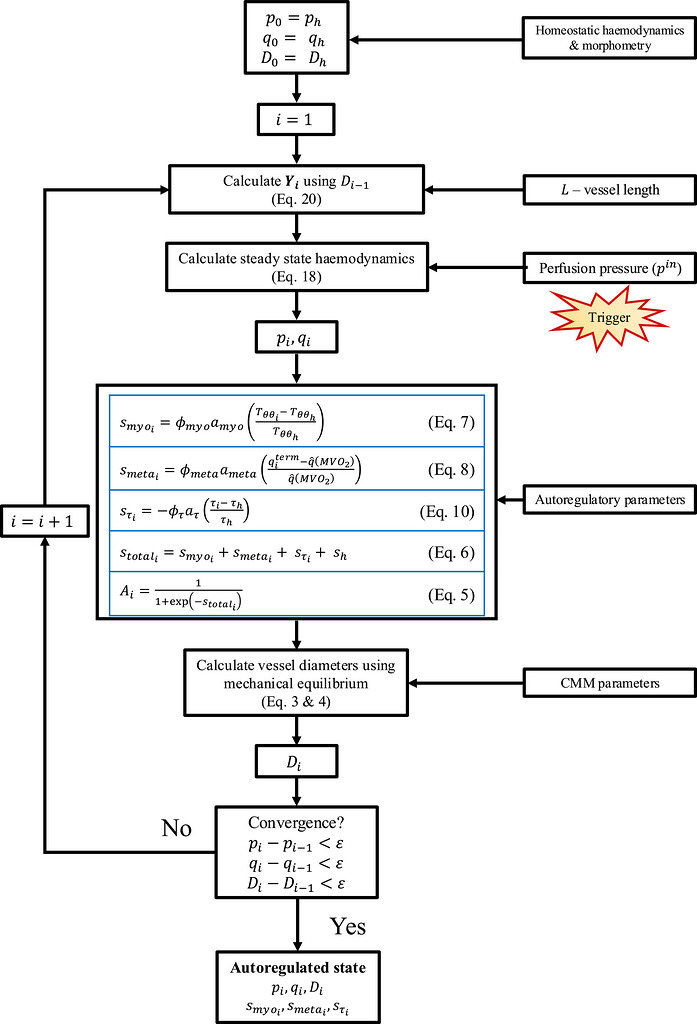
Procedure for calculating an autoregulated state Iterative procedure to solve for the steady‐state coronary haemodynamics for a given perfusion pressure $p^{\mathrm{in}}$

Given a fully calibrated model (i.e. after completing calibration Stages 1–3), we can predict autoregulated states following a certain trigger to the system. The algorithm to predict these autoregulated states is shown in Fig. 4. Starting with the homeostatic tree morphometry, the admittance Y for the steady component of Womersley's solution (i.e. ω=0) is computed for all vessels (Equation 20). Then, accounting for the trigger [e.g. change in perfusion pressure ($p^{in}$)], the steady state haemodynamics are calculated throughout the coronary tree. The updated haemodynamics alter the autoregulatory stimuli ($s_{myo}$, $s_{meta}$, $s_{\tau }$), and consequently SMC activation A. Next, vessel diameters D throughout the tree are calculated using the updated SMC activation (Equations 3 and 4), resulting in a new tree morphometry. The process of updating tree haemodynamics and vessel diameters is iteratively performed until convergence, determined when p, q and D change less than a small number ε between two successive iterations. This algorithm outputs the autoregulated haemodynamics, tree morphometry and autoregulatory stimuli.

For calculating steady‐state haemodynamics at an autoregulated state, a constant capillary resistance $R_{capillary}$ boundary condition, plus a 20 mmHg terminal coronary venous pressure (Pries et al., 1995), is applied to the outlets. The capillary resistance is computed as: $R_{capillary}=\frac{p_{h}^{term}-20\;mmHg}{q_{h}^{term}}$, where $p_{h}^{term}$ and $q_{h}^{term}$ are the terminal arteriole homeostatic pressure and flow, respectively, obtained in Stage 2 model calibration (see Appendix B).

### Framework calibration

All literature data used for model calibration were obtained from left‐ventricular measurements, predominantly from vessels in the left anterior descending (LAD) perfusion territory. Accordingly, the idealized coronary tree developed in this study is intended to be most representative of an LAD microvascular bed. An outline of the framework calibration is presented in this section and discussed in depth in Appendix B. Briefly, the framework was calibrated in three stages (Fig. 1*B*). In Stage 1, passive and myogenic vessel responses at three myocardial depths were calibrated using experimental myography data across four vessel sizes (Liao & Kuo, 1997), while enforcing physiological constraints, including realistic constituent pre‐stretches and a target homeostatic smooth muscle activation of 0.5. In Stage 2, homeostatic coronary tree morphology and haemodynamics were determined using an extended Murray's law optimization approach (Gharahi et al., 2023). For each myocardial depth, the subtree is symmetrically bifurcating and has 12 generations, with CMM parameters determined in Stage 1 calibration assigned to individual vessels according to their homeostatic diameter $D_{h}$ (Small Arteries: $D_{h}$ > 140 µm, Large Arterioles: 140 ≥ $D_{h\;}$> 80 µm, Intermediate Arterioles: 80 ≥ $D_{h}$> 50 µm, Small Arterioles: 50 ≥ $D_{h}$ µm) and myocardial depth. We used 12‐generation trees because when prescribing literature‐based terminal flow rates of 0.001375, 0.0015 and 0.00165 mm^3^/s for the subepicardial, midwall and subendocardial layers, respectively (see Table B1), the 12‐generation configuration provided the best overall agreement with reported flow–diameter and wall shear stress–diameter distributions (see Fig. 6). Note, tree generations are not consistent with the diameter‐defined Strahler order scheme defined by Kassab et al. (1993). The homeostatic smooth muscle activation ($A_{h}$) was calibrated to achieve target vessel wall thickness while maintaining uniform hoop stress across myocardial layers, consistent with experimental observations of transmural differences in vessel wall thickness (Choy & Kassab, 2009). The optimization incorporated experimental bifurcation exponent (ξ) measurements to capture size‐dependent heterogeneity in vessel branching patterns throughout the coronary circulation. Lastly, Stage 3 calibration determined the metabolic ($a_{meta}$) and shear‐dependent ($a_{\tau }$) scaling coefficients using experimental pressure–flow autoregulation data while holding myogenic strength constant. Nelder–Mead optimization minimized the error ($E_{3}$ in Equation B3) between model‐predicted and experimental normalized flow responses across different myocardial depths and perfusion pressures.

### Model sensitivity

A sensitivity analysis is performed to assess model parameter sensitivity using the approach of Pradhan et al. (2016). Three indices, *X_1_*, *X_2_* and *X_3_*, were created to quantify the output sensitivity of calibration Stages 1, 2 and 3, respectively. *X_1_* and *X_3_* were defined as the percentage change in the error functions $E_{1}$ (defined in Appendix B Equation B2) and $E_{3}$ (defined in Appendix B Equation B3), respectively, resulting from a 10% change in individual parameter values:

(23) $$X_{1}=\frac{100}{E_{1}\left(\zeta \right)}\;\left|E_{1}\left(\zeta +0.1\zeta \right)-E_{1}\left(\zeta \right)\right|,$$

(24) $$X_{3}=\frac{100}{E_{3}\left(\zeta \right)}\;\left|E_{3}\left(\zeta +0.1\zeta \right)-E_{3}\left(\zeta \right)\right|,$$

where ζ is the parameter value and $E_{1}\left(\zeta \right)$ and $E_{3}\left(\zeta \right)$ are the associated errors. Because the primary output of Stage 2 calibration is vessel diameters, rather than the value of the cost function used for homeostatic optimization (Appendix D Equation D6), we define the *X_2_* sensitivity metric as:

(25) $$X_{2}=\frac{100}{N_{gen}}\sum_{i=1}^{N_{gen}}\frac{\left|D_{i}\left(\zeta +0.1\zeta \right)-\;D_{i}\left(\zeta \right)\right|}{D_{i}\left(\zeta \right)},$$

where $N_{gen}$ is the total number of generations and $D_{i}$ and is the diameter of generation i. Equation (25) calculates the average change in homeostatic diameter in response to a 10% change in parameter values. Note, the $X_{2}$ metric quantifies the impact of parameter perturbations on the established homeostatic tree, rather than modifying the input parameters during the Stage 2 calibration process itself. We assessed the sensitivity of Stage 2 outputs to material inputs and found the resulting tree morphology to be highly robust, with vessel diameters across all generations exhibiting <0.1% change.

### Ethical statement

This present study is a secondary analysis of previously published data; therefore, additional ethical approval was not required. The primary studies utilized for model development and calibration include Liao and Kuo (1997), Guo and Kassab (2004), Kaimovitz et al. (2008) and Dick et al. (2018). While Guo and Kassab (2004) explicitly state compliance with national and local ethical guidelines, the remaining studies do not include a formal ethics statement. However, these studies were supported by NIH funding and were thus required to adhere to the NIH Guide for the Care and Use of Laboratory Animals and undergo oversight by their respective Institutional Animal Care and Use Committees (IACUC).

## Results

In this section, we present results from model calibration and demonstrate its application through several examples.

**Model calibration and homeostatic predictions**. First, in the Framework calibration section, we review the three calibration stages and compare the passive, active, homeostatic and autoregulatory properties of our coronary tree to literature data. In the Homeostatic coronary flow simulations section, we present homeostatic pulsatile haemodynamic simulations at three myocardial depths and in a small artery and small arteriole.

**Autoregulatory responses to changes in perfusion pressure**. The Autoregulation in response to changes in perfusion pressure section showcases the autoregulatory responses to changes in coronary perfusion pressure, exploring the relative magnitudes of autoregulatory stimuli. In addition, we compare the transmural autoregulatory behaviour and vessel diameter changes with experimental data not used in model calibration.

**Sensitivity studies**. In the Assessing the relative contributions of the autoregulatory mechanisms section, relative contributions of the three autoregulatory mechanisms in coronary pressure–flow autoregulation are examined. Finally, in the section Assessing the impact of vascular dysfunction on autoregulation, we assess how changes in passive and active vessel mechanics affect coronary pressure–flow autoregulation.

### Framework calibration

#### Stage 1 calibration: passive and myogenic response

**Figure 5:**
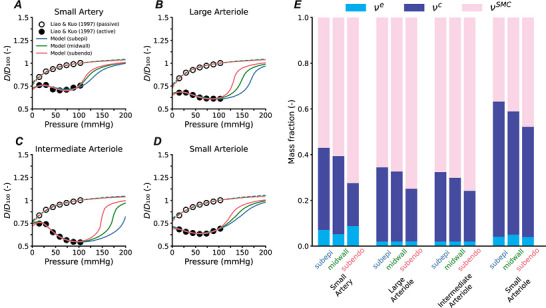
Calibrated pressure‐diameter responses and constituent mass fractions (Stage 1 calibration) Calibrated passive and myogenic responses across four sizes of coronary vessels [*A*: Small Artery ($D_{h}$ = 180 µm), *B*: Large Arteriole ($D_{h}$ = 95 µm), *C*: Intermediate Arteriole ($D_{h}$ = 62 µm), *D*: Small Arteriole ($D_{h}$ = 37 µm)], compared to experimental data (Liao & Kuo, 1997). The *y*‐axis shows diameter normalized by the passive diameter at 100 mmHg ($D_{h}$/$D_{100}$). *E*, estimated mass fractions of elastin ($\nu^{e}$), collagen ($\nu^{c}$), and smooth muscle cells ($\nu^{SMC}$) vary across vessel sizes and myocardial depths.

Our calibrated vessel wall model effectively captures experimentally measured passive and myogenic pressure–diameter relationships across all vessel sizes and myocardial depths (Liao & Kuo, 1997) (Fig. 5*A*–*D*). Calibrated model parameters are given in Tables 1, 2, 3 and Fig. 5*E*. The passive responses (dashed lines) are independent of myocardial depth, whereas the myogenic responses (continuous lines) exhibit clear depth‐dependent differences at $p^{tv}$ > 100 mmHg (Fig. 5*A*–*D*). For each vessel class, the subendo layer has the greatest mass fraction of SMCs (Fig. 5*E*). Notably, large and intermediate arterioles have the largest SMC mass fractions, consistent with having the most pronounced myogenic response (Liao & Kuo, 1997).

**Table 1:** Constrained mixture model material parameters that are constant across vessel sizes

Parameter	Description	Unit	Value	$X_{1}$ Sensitivity (%)			$X_{2}$ Sensitivity (%)			$X_{3}$ Sensitivity (%)	Source
				Subepi	Midwall	Subendo	Subepi	Midwall	Subendo		
$c_{1}$	Elastin material parameter	Pa/kg	94.5	1.0	0.8	0.4	<0.1	<0.1	<0.1	1.4	Calibrated (Stage 1)
$c_{2}$	Collagen material parameter	Pa/kg	308.6	24.2	24.9	11.3	0.4	0.4	0.3	0.2	Calibrated (Stage 1)
$c_{3}$	Collagen dimensionless material parameter	–	3.9	9.5	21.6	16.5	0.1	0.1	<0.1	0.6	Calibrated (Stage 1)
$c_{4}$	SMCs material parameter	Pa/kg	57.2	12.4	13.9	8.2	0.1	0.1	0.3	0.3	Calibrated (Stage 1)
$c_{5}$	SMCs dimensionless material parameter	–	0.59	<0.1	0.4	0.3	<0.1	<0.1	<0.1	0.7	Calibrated (Stage 1)
$S_{\max}$	SMC maximum stress	kPa	250	90.8	135.5	134.1	4.0	4.0	4.3	9.5	(Rachev & Hayashi, 1999; Baek et al., 2007)
$\lambda_{0}$	Zero active tension stretch	–	0.4	15.8	22.8	15.6	<0.1	<0.1	<0.1	0.1	(Rachev & Hayashi, 1999; Baek et al., 2007)
$\lambda_{M}$	Maximum active tension stretch	–	1.1	87.2	123.7	107.0	0.8	0.8	0.8	3.2	(Rachev & Hayashi, 1999; Baek et al., 2007)
$\rho_{\mathrm{wall}}$	Vessel density	kg/m^3^	1060	–	–	–	–	–	–	–	–
$L_{0}$	Metabolic control characteristic decay length	m	0.001	–	–	–	–	–	–	–	(Namani et al., 2018)

**Table 2:** Constrained mixture model autoregulatory parameters that are constant across vessel sizes

Parameter	Description	Unit	Value	$X_{1}$ Sensitivity (%)	$X_{2}$ Sensitivity (%)	$X_{3}$ Sensitivity (%)	Source
		Subepi					
$a_{\mathrm{myo}}$	Myogenic scaling coefficient	–	6.32	101.4	–	<0.1	Calibrated (Stage 1)
$a_{\mathrm{meta}}$	Metabolic scaling coefficient	–	2.82	–	–	0.9	Calibrated (Stage 3)
$a_{\tau }$	Shear‐dependent scaling coefficient	–	1.58	–	–	0.7	Calibrated (Stage 3)

		Midwall					
$a_{\mathrm{myo}}$	Myogenic scaling coefficient	–	5.43	99.6	–	0.7	Calibrated (Stage 1)
$a_{\mathrm{meta}}$	Metabolic scaling coefficient	–	2.82	–	–	0.9	Calibrated (Stage 3)
$a_{\tau }$	Shear‐dependent scaling coefficient	–	1.58	–	–	0.7	Calibrated (Stage 3)

		Subendo					
$a_{\mathrm{myo}}$	Myogenic scaling coefficient	–	4.37	50.8	–	<0.1	Calibrated (Stage 1)
$a_{\mathrm{meta}}$	Metabolic scaling coefficient	–	2.82	–	–	0.9	Calibrated (Stage 3)
$a_{\tau }$	Shear‐dependent scaling coefficient	–	1.58	–	–	0.7	Calibrated (Stage 3)

**Table 3:** Constrained mixture model parameters that are variable across vessel sizes

Small artery: SA, large arteriole: LAo, intermediate arteriole: IAo, and small arteriole: SAo.																		
Parameter	Description	Unit	Value				$X_{1}$ Sensitivity (%)				$X_{2}$ Sensitivity (%)				$X_{3}$ Sensitivity (%)			
			SA	LAo	IAo	SAo	SA	LAo	IAo	SAo	SA	LAo	IAo	SAo	SA	LAo	IAo	SAo
															Subepi			
$G_{\theta }^{e},G_{z}^{e}$	Elastin pre‐stretch	–	1.00	1.29	1.13	1.16	17.6	3.0	2.2	7.3	0.4	<0.1	0.1	0.2	0.2	<0.1	<0.1	2.5
$G_{h}^{c}$	Collagen pre‐stretch	–	1.10	1.02	1.00	1.00	170.1	74.0	73.8	113.8	4.7	0.2	0.1	2.7	1.1	0.3	0.4	36.7
$G_{h}^{m}$	SMCs pre‐stretch	–	1.00	1.00	1.00	1.00	11.6	38.8	51.9	20.1	1.5	0.1	0.1	0.6	0.2	0.2	0.3	7.7
$\upsilon^{e}$	Elastin mass fraction	–	0.07	0.02	0.02	0.04	1.3	0.2	0.3	1.2	<0.1	<0.1	<0.1	<0.1	0.1	<0.1	<0.1	0.1
$\upsilon^{c}$	Collagen mass fraction	–	0.36	0.32	0.30	0.59	17.4	18.4	6.8	78.6	0.4	<0.1	<0.1	<0.1	0.2	0.1	<0.1	2.6
$\upsilon^{m}$ ^*^	SMCs mass fraction	–	0.57	0.66	0.68	0.37	–	–	–	–	–	–	–	–	–	–	–	–
$\phi_{\mathrm{myo}}$	Normalized myogenic strength	–	0.70	0.60	1.00	0.86	–	–	–	–	–	–	–	–	–	–	–	–
$\phi_{\tau }$	Normalized myogenic strength	–	0.62	1.0	0.83	0.42	–	–	–	–	–	–	–	–	–	–	–	–
															Midwall			
$G_{\theta }^{e},G_{z}^{e}$	Elastin pre‐stretch	–	1.01	1.33	1.12	1.07	14.6	2.5	1.7	17.0	0.3	<0.1	0.1	0.3	0.9	0.7	0.7	3.8
$G_{h}^{c}$	Collagen pre‐stretch	–	1.10	1.04	1.00	1.00	168.3	106.5	93.2	132.5	4.6	0.2	<0.1	2.3	1.9	1.0	1.0	32.4
$G_{h}^{m}$	SMCs pre‐stretch	–	1.00	1.02	1.00	1.00	20.6	44.7	62.6	31.1	1.7	0.1	0.1	0.6	0.9	0.9	1.0	8.7
$\upsilon^{e}$	Elastin mass fraction	–	0.05	0.02	0.02	0.05	1.1	<0.1	0.2	3.5	<0.1	<0.1	<0.1	<0.1	0.6	0.7	0.7	0.6
$\upsilon^{c}$	Collagen mass fraction	–	0.34	0.30	0.28	0.54	26.5	20.3	8.5	77.1	0.4	<0.1	<0.1	<0.1	0.5	0.6	0.6	1.6
$\upsilon^{m}$ ^*^	SMCs mass fraction	–	0.61	0.67	0.70	0.41	–	–	–	–	–	–	–	–	–	–	–	–
$\phi_{\mathrm{myo}}$	Normalized myogenic strength	–	0.84	0.70	1.00	0.75	–	–	–	–	–	–	–	–	–	–	–	–
$\phi_{\tau }$	Normalized myogenic strength	–	0.62	1.0	0.83	0.42	–	–	–	–	–	–	–	–	–	–	–	–
															Subendo			
$G_{\theta }^{e},G_{z}^{e}$	Elastin pre‐stretch	–	1.00	1.37	1.20	1.12	22.2	2.4	1.7	10.9	0.5	<0.1	<0.1	0.2	0.3	<0.1	<0.1	2.2
$G_{h}^{c}$	Collagen pre‐stretch	–	1.10	1.03	1.00	1.00	72.9	79.5	81.6	98.2	3.4	0.2	0.1	1.9	0.6	0.2	0.3	24.9
$G_{h}^{m}$	SMCs pre‐stretch	–	1.07	1.03	1.00	1.00	32.4	44.1	57.4	31.3	2.8	0.2	0.1	0.7	0.4	0.2	0.3	7.5
$\upsilon^{e}$	Elastin mass fraction	–	0.09	0.02	0.02	0.04	2.4	0.3	0.2	0.9	<0.1	<0.1	<0.1	<0.1	0.1	<0.1	<0.1	0.1
$\upsilon^{c}$	Collagen mass fraction	–	0.19	0.23	0.22	0.48	7.7	11.0	5.5	52.3	0.3	<0.1	<0.1	<0.1	0.1	<0.1	<0.1	1.1
$\upsilon^{m}$ ^*^	SMCs mass fraction	–	0.72	0.75	0.76	0.48	–	–	–	–	–	–	–	–	–	–	–	–
$\phi_{\mathrm{myo}}$	Normalized myogenic strength	–	0.92	0.74	1.00	0.66	–	–	–	–	–	–	–	–	–	–	–	–
$\phi_{\tau }$	Normalized myogenic strength	–	0.62	1.0	0.83	0.42	–	–	–	–	–	–	–	–	–	–	–	–

* $\upsilon^{m}=1-\upsilon^{e}-\;\upsilon^{c}$

#### Stage 2 calibration: homeostatic morphometry, structure and haemodynamics

**Figure 6:**
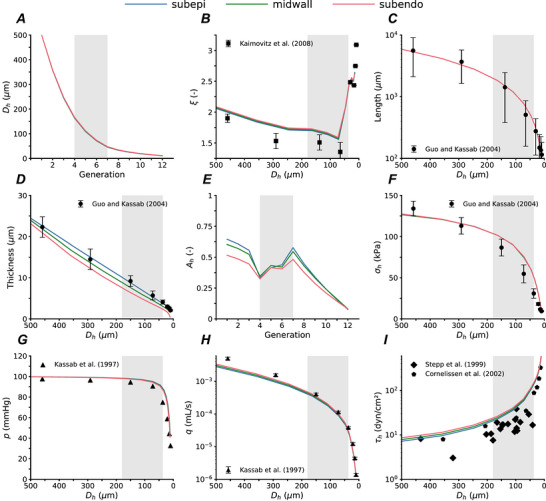
Comparison of coronary trees generated through the homeostatic optimization framework (Stage 2 calibration) The three tree morphologies have little difference in their diameter *vs*. generation (*A*), diameter exponent *vs*. diameter (*B*) and vessel length *vs*. diameter (*C*) distributions. However, there is a layer dependence on vessel thickness (*D*). The homeostatic SMC activation (*E*) was found to vary between the three layers when targeting equivalent hoop stress distributions (*F*). Mean pressure (*G*), flow (*H*) and shear stress (*I*) vary slightly because of different terminal flows being prescribed for the three tree layers. The grey box indicates vessel diameters in the Liao and Kuo (1997) dataset. Literature data are presented as mean ± SD.

Homeostatic optimization results are summarized in Fig. 6. Vessel diameters range from 11 to 500 µm across 12 tree generations and show minimal dependence on myocardial depth (Fig. 6*A*). Consequently, diameter bifurcation exponent ξ is also not depth dependent (Fig. 6*B*) and is qualitatively aligned with LAD measurements by Kaimovitz et al. (2008), though our model predicts larger ξ exponents for *D* > 65 µm. The vessel length–diameter relationship matches LAD data presented by Guo and Kassab (2004) and does not differ between subtrees (Fig. 6*C*).

Subepi tree thickness (blue line in Fig. 6*D*) was calibrated using LAD experimental data by Guo and Kassab (2004) through calibration of the SMC homeostatic activation parameter $A_{h}$ (Fig. 6*E*). This produced a hoop stress distribution, which was then assumed to be identical across all three subtrees (Fig. 6*F*). Using this assumption, distributions of thickness and SMC homeostatic activation $A_{h}$ could be determined for the midwall and subendo trees (green and red lines in Fig. 6*D* and *E*, respectively). Subepi vessels are thicker than subendo vessels, consistent with anatomical measurements in the left ventricle (Choy & Kassab, 2009). $A_{h}$ was highest in large vessels and lowest in small vessels (Fig. 6*E*). Of note, the vessel sizes in the Liao and Kuo (1997) dataset used in Stage 1 calibration span generations 4–7, where noticeable changes in $A_{h}$ occur. Additionally, $A_{h}$ was lowest in the subendo layer, while the subepi and midwall layers exhibited similar activation levels. The hoop stress distributions across all three coronary layers demonstrate fair agreement with Guo and Kassab (2004) LAD measurements (Fig. 6*F*).

Pressure and flow distributions are presented in Fig. 6*G* and *H*, respectively, and are comparable to the left common coronary artery pressure and flow distributions calculated by Kassab et al. (1997). Most of the vascular resistance is in arterioles with $D_{h}$ < 100 µm, as shown by the rapid decrease in pressure in Fig. 6*G*. Wall shear stress increases roughly 80‐fold across the coronary trees (Fig. 6*I*), with values calculated by our model tending to be greater than that reported by Stepp et al. (1999) in canine left ventricle epicardial vessels, but in fair agreement with the mathematical model presented by Cornelissen et al. (2002) based on porcine data. Collectively, Fig. 6 shows that our homeostatic optimization framework produced a coronary tree that is morphologically and haemodynamically consistent with what has previously been reported in the literature.

#### Stage 3 calibration: coronary pressure–flow response

**Figure 7:**
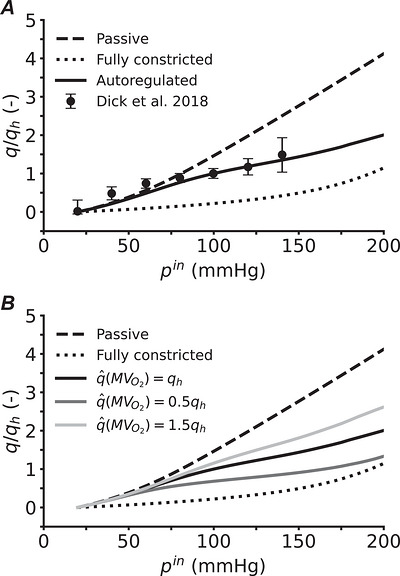
Calibrated pressure‐flow response (Stage 3 calibration) *A*, the calibrated autoregulated pressure–flow relation for the entire tree (continuous line) compared to experimental measurements (Dick et al., 2018), with $q_{h}$ being the homeostatic flow rate. *B*, a 50% increase or decrease in myocardial metabolic demand shifts the coronary pressure–flow relationship vertically up or down, respectively. Literature data are presented as mean ± SD.

Calibrated values of metabolic $a_{meta}$ and shear‐dependent $a_{\tau }$ scaling coefficients are presented in Table 2. Furthermore, the autoregulated, fully dilated (A=0) and maximally constricted (A=1) flow–pressure relationships are presented in Fig. 7*A*, with the ratio of total flow (*q*) over homeostatic flow (*q_h_*) in the coronary arteries plotted over varying inlet perfusion pressures $p^{in}$. At $p^{in}\in \left(80-140\right)$ mmHg, our model fits the experimentally reported flow–pressure relationship well. However, our model underpredicts flow at $p^{in}$ of 40 and 60 mmHg. Note, in this pressure range the predicted autoregulation curve is closely aligned to the fully dilated pressure–flow relation, indicating a full dilatation of the resistive vessels. To assess how our model responds to changes in metabolic demand, we computed flow–pressure relationships in response to a 50% increase $\left[\hat{q}\left(\mathrm{MV}O_{2}\right)=1.5q_{h}\right]$ and 50% decrease $\left[\hat{q}\left(\mathrm{MV}O_{2}\right)=0.5q_{h}\right]$ in target coronary flow (see Equation 8). As expected, the flow–pressure curves show upwards and downwards shifts in response to increases and decreases in metabolic demands, respectively (see Fig. 7*B*).

### Homeostatic coronary flow simulations

**Figure 8:**
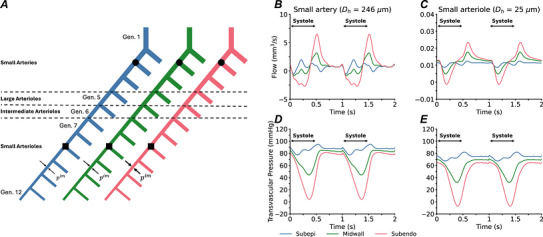
Coronary homeostatic haemodynamics Graphical representation of the three coronary subtrees (*A*). The circular and square markers indicate the location on the subtrees from wherethe pulsatile flow (*B*, *C*) and transvascular pressures (*D*, *E*) are taken.

Coronary flow and transvascular pressure waveforms are shown for a small artery ($D_{h}$ = 246 µm, circular marker in Fig. 8*A*) and small arteriole ($D_{h}$ = 25 µm, square marker in Fig. 8*A*) for subepi, midwall and subendo subtrees. Flow and pressure pulsatility is greatest in the subendo layer for both vessel sizes (Fig. 8*B*–*E*), attributable to the intramyocardial pressure ($p^{im}$) waveform having the greatest pulse pressure in the subendo layer (Fig. 3*A*). Flow is diastolically dominant for both vessel sizes in the midwall and subendo subtrees. However, blood flow is more evenly distributed throughout the cardiac cycle in the subepi layer, with 53.3% and 47.8% of total flow occurring during systole for the small artery and small arteriole, respectively (Fig. 8*B* and *C*). Retrograde flow occurs in all subtrees for the small artery, with the subendo layer having the most retrograde flow and the subepi layer having the least (Fig. 8*B*). Conversely, the small arteriole exhibits no retrograde flow in the subepi and midwall layers, and only minimal retrograde flow in the subendo layer (Fig. 8*C*). Notably, the shape of the subepi transvascular pressure waveforms differs from the midwall and subendo waveforms during systole and early diastole (Fig. 8*D* and *E*), which ultimately impacts flow dynamics.

### Autoregulation in response to changes in perfusion pressure

#### Pressure, diameter, wall shear stress and autoregulatory stimuli

**Figure 9:**
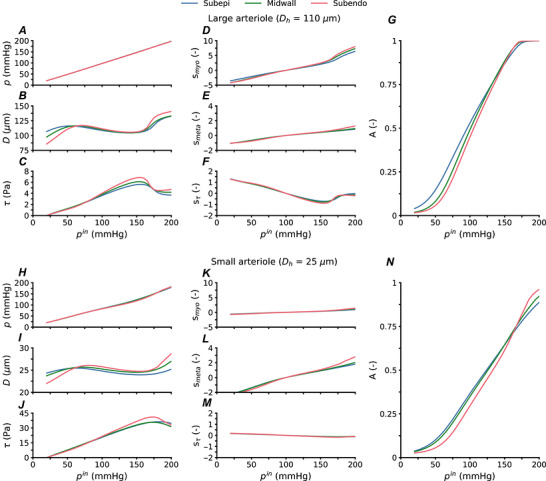
Pressure‐flow autoregulatory response for a large and small arteriole In the large arteriole, lumen pressure increases linearly (*A*), whereas vessel diameter (*B*) and wall shear stress (*C*) exhibit non‐monotonic responses to changing coronary perfusion pressure ($p^{in}$). The myogenic stimulus (*D*) is greater than the metabolic (*E*) and shear‐dependent (*F*) stimuli, indicating myogenic control plays a dominant autoregulatory role in the large arteriole. SMC activation curve (*G*) is steepest in the subendo layer and shallowest in the subepi layer, indicative of a layer‐dependent autoregulatory range. In the small arteriole, lumen pressure increases non‐linearly (*H*), and vessel diameter (*I*) and wall shear stress (*J*) exhibit a response qualitatively similar to the larger arteriole. Contrary to the large arteriole, the small arteriole myogenic stimulus (*K*) is weak compared to the metabolic stimulus (*L*). Shear‐dependent control plays a negligible role in the small arterioles (*M*). The small arteriole SMC activation curve (*N*) exhibits a layer‐dependent response.

Pressure, diameter, wall shear stress, autoregulatory stimuli and SMC activation (A) are presented for varying perfusion pressures in a large arteriole ($D_{h}$ = 110 µm; Fig. 9*A*–*G*) and small arteriole ($D_{h}$ = 25 µm; Fig. 9*H*–*N*).

Lumen pressure (p) increases linearly with perfusion pressure ($p^{in}$) in the large arteriole (Fig. 9*A*) and a slight non‐linear pattern in the small arteriole (Fig. 9*H*). For both vessel sizes, p shows minimal variation between the layers. Diameter (D) responses (Fig. 9*B* and *I*) show both vessel sizes modulating their diameters over the autoregulatory pressure range, with vessel diameters decreasing as $p^{in}$ increases from approximately 60 to 165 mmHg. Outside this range, diameters increase with increasing pressure, consistent with the mechanical behaviour in vessels not actively modulating tone. Notable differences in the diameter–pressure response between the three layers are apparent, with the subendo layer actively modulating its diameter over the narrowest range of $p^{in}$. Wall shear stress (τ) increases with perfusion pressure in both vessel sizes until approximately 165 mmHg before declining (Fig. 9*C* and *J*), which is consistent with the sharp increase in diameter. The change in wall shear stress is layer‐dependent; however, it is qualitatively consistent between the layers.

Next, we examine the relative contribution to SMC activation A of the myogenic, metabolic and shear‐dependent stimuli. All stimuli are set to zero at the homeostatic perfusion pressure (here, $p^{in}$= 100 mmHg; see Equations 7, 8 and 10). Larger deviations from zero therefore indicate larger contributions from that signal. The large arteriole exhibits predominantly myogenic control (Fig. 9*D*), with the myogenic stimulus lowest in the subendo layer for $p^{in}$ < 100 mmHg and greatest for $p^{in}$ > 100 mmHg. Metabolic and shear‐dependent contributions are comparatively small and show minimal layer dependence (Fig. 9*E* and *F*). In contrast, the small arteriole shows greater metabolic control (Fig. 9*L*), with myogenic control contributing substantially less than in the large arteriole (Fig. 9*K*). In small arterioles, the metabolic stimulus is lowest in the subendo layer for $p^{in}$ < 100 mmHg and greatest for $p^{in}$ > 100 mmHg, whereas myogenic and shear‐dependent stimuli remain relatively uniform across layers (Fig. 9*K* and *M*). These differences in stimulus between the vessels are a direct consequence of the assumed normalized response curves in Fig. 2*A*. Lastly, Fig. 9*G* and *N* show the relationships between SMC activation A and $p^{in}$. The steepest patterns are found in the subendo layer for both vessel sizes, indicating that vessels in this layer reach maximum dilatation (A = 0) or constriction (A = 1) within a narrower pressure range.

#### ENDO/EPI responses

**Figure 10:**
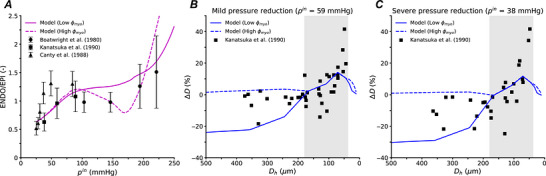
Model responses to varying inlet perfusion pressures ($p^{\mathrm{in}}$) *A*, the calculated ratio of flow going into the subepi layer *vs*. subendo layer (ENDO/EPI) matches the trends reported in the literature. Both Low and High $\phi_{myo}$ models show dilatation (Δ*D* > 0) in vessels with a homeostatic diameter ($D_{h}$) < 160 µm in response to a mild (*B*) and severe (*C*) reduction in $p^{in}$. For vessels with a $D_{h}$> 160 µm, the Low $\phi_{myo}$ model predicts constriction, while the High $\phi_{myo}$ model results in Δ*D* remaining nearly unchanged. The grey box indicates vessel diameters in the dataset of Liao and Kuo (1997). Literature data are presented as mean ± SD.

The ratio of subendo to subepi flow (ENDO/EPI) at varying perfusion pressures is studied next, using data never used for the calibration of our model. Figure 10*A* shows ENDO/EPI data in both anaesthetized (Boatwright et al., 1980; Kanatsuka et al., 1990) and un‐anaesthetized (Canty, 1988) canine experiments in the left ventricle. We run our model with two different assumptions for the normalized myogenic response curve (Low and High $\phi_{myo}$, Fig. 2). A homeostatic ENDO/EPI ratio of 1.2 at $p^{in}$ = 100 mmHg is enforced by setting the terminal arterioles outflow ratio: $q_{subendo}^{term}/q_{subepi}^{term}$ = 0.00165/0.001375, see Table B1. From this homeostatic setpoint, the Low $\phi_{myo}$ profile produces increased ENDO/EPI ratios for $p^{in}$ > 100 mmHg, and decreased ENDO/EPI ratios for $p^{in}$ < 100 mmHg. This trend is consistent with the canine experimental data. Conversely, the High $\phi_{myo}$ myogenic profile reveals profound differences on ENDO/EPI ratio for $p^{in}$ > 100 mmHg, but not for $p^{in}$ < 100 mmHg.

#### Diameter responses

Next, we explore changes in subepi vessel diameters in response to mild ($p^{in}=$ 59 mmHg, Fig. 10*B*) and severe ($p^{in}=$ 38 mmHg, Fig. 10*C*) reductions in perfusion pressure. Percentage change in vessel diameter (Δ*D*) is compared to experiments in canine left ventricle epicardial vessels (Kanatsuka et al., 1990) using the Low and High $\phi_{myo}$ profiles (see Fig. 2). Using the Low $\phi_{myo}$ profile, for both reductions in perfusion pressure, vessels with $D_{h}$ < 160 µm dilate while vessels with $D_{h}$ > 160 µm constrict (Fig. 10*B* and *C*). In contrast, the High $\phi_{myo}$ response produces similar dilatations for vessels $D_{h}$ < 160 µm, but relatively unchanged Δ*D* for vessels $D_{h}$ > 160 µm for both reductions in perfusion pressure (Fig. 10*B* and *C*). This suggests that differences between Low and High $\phi_{myo}$ models are greatest for the larger vessels.

### Assessing the relative contributions of the autoregulatory mechanisms

**Figure 11:**
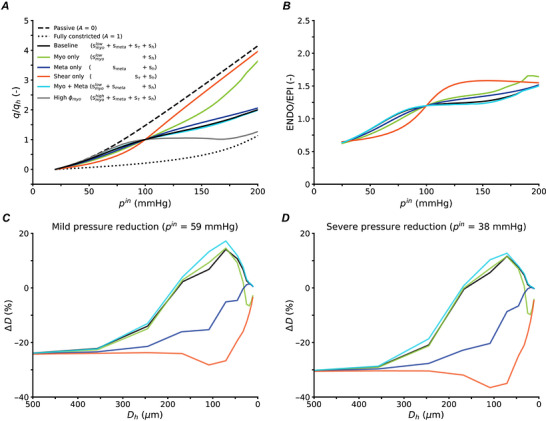
Simulated contributions of the autoregulatory mechanisms Impact of autoregulatory mechanisms on (*A*) the coronary pressure–flow relationship, (*B*) transmural flow distribution (ENDO/EPI) and subepi vascular diameter responses to (*C*) mild and (*D*) severe reductions in perfusion pressure. High $\phi_{myo}$ is only plotted in *A*, as it is included in Fig. 10.

In Fig. 11*A*, we explore the impact on pressure–flow response of six different combinations of autoregulatory mechanisms (see Equation 6). In all six combinations, the basal homeostatic stimulus ($s_{h}$) is maintained. The passive (*A* = 0), fully constricted (*A* = 1) and baseline autoregulated response curves from Fig. 7*A* are also presented. The metabolic‐only case (i.e. with $s_{myo}=\;s_{\tau }=0$) yields a greater autoregulatory response compared to the myogenic‐ or shear‐dependent‐only cases. The myogenic‐only case maintains some autoregulatory capacity, whereas the shear‐dependent‐only case does not autoregulate. For the myo + met case, the changes in coronary autoregulation are minor compared to the baseline autoregulated case, emphasizing that shear‐dependent control has minimal contributions to pressure–flow autoregulation. These results suggest that the relative contributions of the autoregulatory mechanisms follow the order: metabolic > myogenic > shear‐dependent. Lastly, when replacing the Low $\phi_{myo}$ with the High $\phi_{myo}$ distribution (Fig. 2*B*), myogenic control substantially strengthens the pressure–flow autoregulatory response. Therefore, these responses are greatly dictated by the assumed normalized autoregulatory strength distributions.

The impact of autoregulatory mechanisms on transmural flow distribution (ENDO/EPI) (Fig. 11*B*) reveals that the myo‐only, meta‐only and myo + meta cases exhibit behaviour qualitatively consistent with the baseline case across perfusion pressures. Conversely, the ENDO/EPI ratio in the shear‐only case exhibits a distinct behaviour, characterized by high sensitivity to minor fluctuations in perfusion pressure around the homeostatic pressure. This suggests that shear‐dependent mechanisms alone are insufficient for regulating transmural flow distribution.

Mild ($p^{in}=$ 59 mmHg, Fig. 11*C*) and severe ($p^{in}=$ 38 mmHg, Fig. 11*D*) reductions in perfusion pressures produce changes in subepi vessel diameters (Δ*D*) that are qualitatively similar to each other for all combinations of autoregulatory mechanisms considered. For $D_{h}$ > 160 µm, the myo‐only and myo + meta cases closely follow the baseline curve, whereas the meta‐only and shear‐only cases experience greater constriction. This is indicative of myogenic control being the most influential in determining the vascular response for vessels $D_{h}$ > 160 µm. For vessels 50 < $D_{h}$ < 160 µm, the myo + meta case experiences the greatest dilatation, due to the absence of the constrictive stimulus provided by the shear‐dependent mechanism. Conversely, the shear‐only case demonstrates the most pronounced constriction, as the reduction in perfusion pressure leads to a decrease in flow (Fig. 11*A*) and a consequent loss of shear‐mediated dilatory stimulus. The myo‐only case best follows the baseline curve in the 50 < $D_{h}$ < 160 µm range, also indicative of the myogenic response being the dominant mechanism in this diameter range. However, for $D_{h}$ < 50 µm, the myo‐only case becomes more constricted than the baseline and converges toward the shear‐only curve. By contrast, both the meta‐only and myo + meta cases converge with the baseline case in this diameter range. This behaviour indicates that the metabolic mechanism is the dominant regulator for the smallest vessels, with myogenic and shear‐dependent mechanisms playing a diminished role.

### Assessing the impact of vascular dysfunction on autoregulation

**Figure 12:**
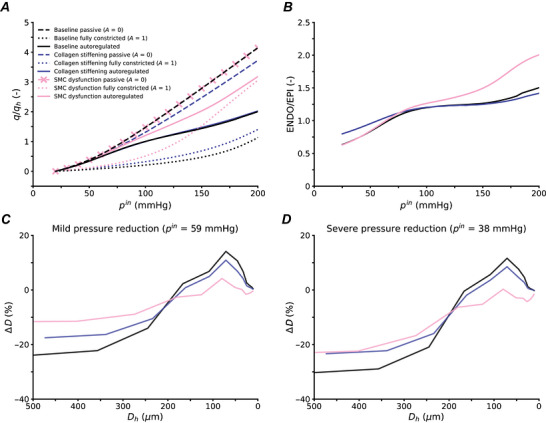
Impact of collagen stiffening and smooth muscle cell dysfunction on autoregulation Analysis on how changes in vascular mechanical properties impact (*A*) coronary pressure–flow relationships, (*B*) transmural flow distribution (ENDO/EPI), and vascular diameter responses to (*C*) mild and (*D*) severe reductions in perfusion pressure.

A key benefit of a microstructurally motivated model of coronary autoregulation is its integration of microstructural properties and cellular‐level functions within the vessel wall model, enabling investigation into how structural and cellular changes affect coronary circulation function. We demonstrate the utility of our framework by examining two scenarios of microstructural remodelling: (1) stiffening of collagen fibres and (2) impaired SMC contractility. Specifically, collagen fibres are stiffened by doubling the passive material parameter $c_{2}$, while SMC contractility is impaired by halving $S_{max}$ (see Table 1). The effects of these two scenarios on flow–pressure autoregulation are shown in Fig. 12*A*. For reference, the passive (*A* = 0), fully constricted (*A* = 1) and baseline autoregulated response curves from Fig. 7*A* are also presented.

Stiffening of the collagen fibres results in upward and downward shifts of the fully constricted and dilated curves, respectively. However, coronary autoregulation remains relatively intact, suggesting that collagen stiffening constrains the dilatory capacity of the coronary tree without abolishing its autoregulatory function. In contrast, impaired SMC contractility produces a loss of coronary autoregulation accompanied by an upward shift in the fully constricted curve (Fig. 12*A*). This indicates that reduced SMC contractile capacity severely compromises the ability of the coronary circulation to autoregulate.

The impact of these mechanical changes on transmural flow distribution (Fig. 12*B*) shows that collagen stiffening produces a flattening of the ENDO/EPI ratio curve following deviations in $p^{in}$ when compared to the baseline. SMC dysfunction causes the ENDO/EPI ratio to increase for $p^{in}$ > 100 mmHg, but shows little change for $p^{in}$ < 100 mmHg. Both collagen stiffening and SMC dysfunction reduce the magnitude of Δ*D* in response to mild (Fig. 12*C*) and severe (Fig. 12*D*) reductions in perfusion pressure. This demonstrates that both vascular dysfunctions limit the ability of the vessel to modulate diameter, as evidenced by the narrowing between the passive and fully constricted curves in Fig. 12*A*. Note, changing the passive and active material properties influences the homeostatic morphology of the tree, as evident by the different homeostatic diameters in Fig. 12*C* and *D*.

## Discussion

The coronary circulation has been the subject of many modelling studies over the years. A major focus has been image‐based simulations of epicardial coronary haemodynamics to quantify local flow patterns and assess the functional significance of epicardial stenoses (Kim et al., 2010; Lissoni et al., 2025; Malvè et al., 2012; Taylor et al., 2013, 2023). These studies are typically calibrated to a single haemodynamic state (e.g. resting or hyperaemic conditions) and often account for the microcirculation using lumped‐parameter models. Recent studies have coupled epicardial vessels to synthetic microvascular networks to simultaneously simulate epicardial and microvascular haemodynamics (Kim et al., 2023; Menon et al., 2024; Papamanolis et al., 2021); however, these models do not yet capture the regulatory functions of the coronary microvascular. The coronary circulation manifests a complex dynamic response to changes in perfusion pressure over multiple timescales. These timescales span from autoregulatory variations in active SMC tone, taking place within minutes, to slower changes in vessel microstructure (e.g. stiffening, SMC dysfunction) (Kassab et al., 2002; Lu et al., 2011; Trask et al., 2012), to vascular network angiogenesis or rarefaction over the course of weeks to months (Pries et al., 2015).

Previous modelling endeavours of coronary autoregulation have focused on capturing key short‐term features using primarily lumped‐parameter approaches (Cornelissen et al., 2002; Gharahi et al., 2022; Namani et al., 2018, 2020; Xie & Wang, 2015). These models lack detailed structural and morphometric information on vascular networks, and are therefore not suitable to predict the long‐term pathophysiological alterations in the coronary circulation noted above. To address this need, this work aimed to produce a unified computational framework integrating vascular responses at the short time scales (e.g. autoregulation) as well as long time scales (e.g. vascular growth and remodelling) while including morphometric, haemodynamics and structural (i.e. vessel wall composition) data (Fig. 1*A*). This design goal resulted in the adoption of a microstructurally motivated modelling framework based on constrained mixture theory, which integrates vessel microstructural properties and cellular‐level functions in a non‐linear continuum mechanics framework. To our knowledge, this is the first implementation of a constrained mixture theory to model coronary autoregulation. The main findings of the model are discussed next.

### CMM parameter estimation

In Stage 1 of model calibration, the vascular wall model was calibrated using passive and myogenic pressure–diameter data from *ex vivo* myography measurements in swine across four vessel sizes (Liao & Kuo, 1997). To apply these data to all myocardial layers, we assumed the passive and myogenic response does not vary transmurally across the myocardium for the transvascular pressures tested experimentally ($p^{\mathrm{tv}}\leq$ 100 mmHg). To date, only a limited number of studies have investigated whether the myogenic response varies transmurally across the myocardium (Essajee et al., 2026; Kuo et al., 1988; Sorop et al., 2008), and it remains inconclusive whether meaningful differences exist (Dick et al., 2018).

Model parameters include passive and active material properties, constituent pre‐stretches and mass fractions (Tables 1, 2, 3). The calibrated model effectively captures the passive and myogenic responses for all vessel sizes, at each myocardial depth (Fig. 5*A*–*D*). For $p^{\mathrm{tv}}>$ 100 mmHg, there are clear differences in the myogenic response between the three subtrees, with the subepi layer experiencing a greater myogenic response compared to the subendo layer. The myogenic response varies between the subtrees partly because vessel wall thickness decreases at greater myocardial depths (Fig. 6*D*), which decreases the total mass of load‐bearing constituents. Future studies probing the myogenic response across a broader range of pressures would elucidate whether myogenic control varies throughout the myocardium.

The calibrated SMC mass fraction is greatest in the subendo layer and lowest in the subepi layer for all vessel sizes (Fig. 5*E*). The subendo layer probably has the greatest SMC mass fraction to compensate for its thinner vessels (Fig. 6*D*), enabling it to generate sufficient active tension. Elastin mass fraction remained <10% for all vessel sizes considered, which aligns with these vessels being muscular in nature. To date, mass fractions of coronary artery constituents have only been quantified in large epicardial arteries (Chen & Kassab, 2016), while the microcirculation remains understudied. Microscopic analysis of rabbit arterioles showed that SMC content gradually decreases from 100 to 30 µm vessels (Rhodin, 1967), consistent with our SMC mass fractions decreasing from intermediate to small arterioles. However, a quantitative assessment of the wall constituents was not performed. Future work quantifying the microstructural composition of the coronary microcirculation will enhance our understanding of coronary physiology and pathophysiology (Hayenga et al., 2012).

### Morphometry, structure and haemodynamics of homeostatic coronary trees

In Stage 2 of model calibration, the homeostatic optimization method proposed by Gharahi et al. (2023) was used to generate three symmetrically bifurcating coronary trees in subepi, midwall and subendo layers. This approach estimates the homeostatic morphometric (e.g. diameters), structural (e.g. thicknesses) and haemodynamic (e.g. pressure and wall shear stress) characteristics of the vascular trees by minimizing the metabolic and viscous energy dissipation under the constraint of mechanical equilibrium. Furthermore, to incorporate bifurcation diameter exponent (ξ) measurements into the homeostatic optimization (Kaimovitz et al., 2008), a penalty term was added into the cost function used in Gharahi et al. (2023). Other approaches for determining a homeostatic state utilize measured or assumed reference geometries and estimate constitutive parameters, including prestretches, to match the prescribed homeostatic geometry (Szafron et al., 2023; Wu et al., 2021). Both approaches are valid and depend on the information available for calibration.

The morphological and homeostatic characteristics of the three subtrees are summarized in Fig. 6. Swine measurements by Guo and Kassab (2004) were used as targets for the subepi wall thickness, whereas the midwall and subendo thicknesses were calibrated to match the subepi hoop stress distribution. The decision to match the hoop stress distributions of the three subtrees was based on the experimental findings that vessel wall thickness varies transmurally across the myocardium (Choy & Kassab, 2009), suggesting that mechanical stimuli play an important role in determining vessel thickness (Choy & Kassab, 2009; Choy et al., 2006).

Vessel thickness was calibrated by tuning SMC homeostatic activation ($A_{h}$). The predicted homeostatic activation level decreases across generations 1–4 and 7–12, while generations 4–7 deviate from this trend (Fig. 6*E*). The decreasing $A_{h}$ across generations 1–4 and 7–12 is probably a consequence of extrapolating constant material properties to vessels outside the diameter range used for vessel wall model calibration (i.e. all small arteries and small arterioles have the same material parameters). The pressure–diameter data from Liao and Kuo (1997) encompass vessel diameters of 35–180 µm, whereas our tree spans diameters 11–500 µm. Using variable material parameters might be more appropriate and aid in generating a vessel‐size‐independent distribution of $A_{h}$ (Szafron et al., 2023), but we chose not to because our current model already contains many parameters and uses non‐linear constitutive models. Our model would benefit from further characterization of active and passive material properties beyond the experimental range utilized in this study.

The discrepancy between our calibrated diameter exponent values for vessels $D_{h}$ > 65 µm (Fig. 6*B*) and those reported by Kaimovitz et al. (2008) is due to the two competing terms in the homeostatic cost function (Equation D6), where the first term represents the extended Murray's law and the second term incorporates the experimental measurements. In isolation, the extended Murray's law yields a diameter exponent of ∼3 across the tree (Gharahi et al., 2023); however, the inclusion of the Kaimovitz et al. (2008) dataset pulls the diameter exponent towards the experimental values. Thus, the calibrated homeostatic diameter exponent represents a balance between the energy‐based Murray's law and the experimental data. Nevertheless, our pressure and flow distributions showed reasonable agreement with previous modelling efforts (Fig. 6*G* and *H*) (Kassab et al., 1997), indicative of our homeostatic optimization producing a physiological distribution of vessel diameter (and consequently resistance) across the trees. Note that the inlet pressures for all subtrees were assumed to be 100 mmHg based on the study by Mittal et al. (2005) that showed that pressure does not significantly drop in vessels of 100–1000 µm. Our wall shear stress tends to be greater than that measured by Stepp et al. (1999) in canines, but agrees better with previous modelling work by Cornelissen et al. (2002) which is calibrated using porcine data. Collectively, Stage 2 calibration produced coronary trees at three myocardial depths which have morphological and haemodynamic characteristics which reasonably agree with those reported in the literature (Fig. 6).

### Pulsatile coronary haemodynamics

Coronary flow simulations were performed by extending Womersley's solution to account for an external time‐varying intramyocardial pressure (Mackenzie, 2021). This approach allows our model to capture important coronary flow physics that are neglected in 0D lumped parameter models, including wave propagations and reflections that influence pressure and flow waveforms (Fig. 8). Furthermore, each vessel has a longitudinal spatial variable, removing the spatial ambiguity inherent in lumped parameter models.

Flow is diastolically dominant for all vessels in the midwall and subendo layer, whereas flow is more evenly split between systole and diastole in the subepi layer (Fig. 8). The subepi layer experiences the lowest $p^{im}$, and thus the lowest impedance due to vessel compression. Capturing pulsatile flow waveforms across the heart is important for understanding the spatial heterogeneity of myocardial perfusion and plays an important role in oxygen delivery (Sturgess et al., 2024). Collectively, our results show that the magnitude and distribution of $p^{im}$ are important influences on coronary flow waveforms across the myocardium. Importantly, by extending Womersley's solution to account for time‐varying intramyocardial pressure, we demonstrate that this framework can model haemodynamics across the cardiac cycle, in addition to autoregulatory (minutes) and growth and remodelling (weeks) timescales, spanning key temporal scales of coronary physiology.

### Coronary autoregulation model

The coronary autoregulation model is inspired by earlier formulations presented in the literature. We used the SMC activation model proposed by Carlson et al. (2008), which modulates SMC tone as a sigmoidal function in response to a net autoregulatory stimulus ($s_{total}$). In addition, we formulated the autoregulatory stimuli in terms of their deviation from a homeostatic value, which was motivated by previous applications of CMMs in the study of long‐term active SMC‐mediated adaptations in cerebral vasospasms (Baek et al., 2007).

The model resulting from Stage 3 calibration effectively captures coronary pressure–flow autoregulation over a pressure range of 80–140 mmHg (Fig. 7*A*); however, deviations from experimental data are apparent at lower perfusion pressures (40–60 mmHg). Over lower perfusion pressures, the model closely follows the fully passive curve, with SMCs being nearly fully dilated (Fig. 9). Since even the fully passive curve falls below the targeted flow levels (Fig. 7*A*), inability to capture the flow–pressure relationship between 40 and 60 mmHg appears related to the passive mechanics of our vessels. As previously mentioned, the passive and myogenic mechanics are determined by *ex vivo* myography data (Liao & Kuo, 1997), which do not capture the impact of myocardial tethering. It has been shown that myocardial tether plays an important role in vessel mechanics (Hamza et al., 2003; Hoffman & Spaan, 1990; Namani et al., 2018), and therefore important for capturing the *in vivo* pressure–diameter response. Future efforts should include myocardial tethering in the vessel wall model.

We explored the response of the model to changes in perfusion pressure. For the two different myogenic response curves considered (Low $\phi_{myo}$ and High $\phi_{myo}$), a reduction in coronary perfusion pressure decreases the ENDO/EPI flow ratio (Fig. 10*A*), consistent with the known susceptibility of the subendo layer to ischaemia during hypotension (Algranati et al., 2011; Canty, 1988; Kanatsuka et al., 1990). Increases in perfusion pressure in the Low $\phi_{myo}$ case resulted in increasing ENDO/EPI ratios, as reported by Boatwright et al. (1980). Thus, the model captures a transmural variability in autoregulatory capacity. Changes in ENDO/EPI are partially a consequence of subendo vessels becoming fully dilated or constricted within a narrower pressure range when compared to subepi vessels, illustrated by the steeper SMC activation curves in subendo vessels (Fig. 9*G* and *N*). Conversely, increases in perfusion pressure in the High $\phi_{myo}$ case resulted in non‐monotonic changes in the ENDO/EPI ratio, demonstrating the impact of the assumed profiles presented in Fig. 2.

We also compared changes in vessel diameters (Δ*D*) in response to mild ($p^{in}$ = 59 mmHg) and severe ($p^{in}$ = 38 mmHg) reductions in coronary perfusion pressure with those reported by Kanatsuka et al. (1990) in canines. In the Low $\phi_{myo}$ case, we found that vessels with $D_{h}$ > 160 µm constricted while $D_{h}$ < 160 µm dilated, in agreement with measurements (Fig. 10*B* and *C*). Using the High $\phi_{myo}$ profile, the greater myogenic response cause vessels with $D_{h}$ > 160 µm to have relatively unchanged diameter in response to a mild and severe reduction in pressure, contrary to the Low $\phi_{myo}$ case. Furthermore, the dilation in vessels $D_{h}$ < 160 µm was found to be similar between the Low and High $\phi_{myo}$ cases, indicative of vessels being fully dilated with both $\phi_{myo}$ profiles. For both the mild and severe pressure reductions, our model showed a maximum dilatation at a $D_{h}$ = 72 µm, and a decreasing dilatation for smaller vessels, a trend that differs from the experimental data reported by Kanatsuka et al. (1990). However, other models such as that of Cornelissen et al. (2002) produced patterns consistent with our deceasing Δ*D* trends for smaller diameters, although their model investigated changes in response to adenosine and l‐NAME infusion in the coronary microcirculation (Jones et al., 1995), rather than changes in response to perfusion pressure. Regardless, it is clear that accurate characterization of this behaviour would require further experimental data on the passive and active mechanics of the smallest coronary arterioles.

### Model sensitivity

The Stage 1 sensitivity analysis reveals the sensitivity ranking of passive material parameters to be *c*_2_ > *c*_3_ > *c*_4_ > *c*_1_ > *c*_5_, when averaged across the three myocardial layers (Table 1). The high sensitivity to collagen parameters (*c*_2_ and *c*_3_) aligns with collagen's highly non‐linear behaviour and its substantial contribution to the vessel wall mass (Fig. 5*E*). The passive SMC parameter *c*_4_ is the third most sensitive, while the SMC non‐linearity parameter *c*_5_ is the least sensitive. Although SMCs contribute minimally to the passive mechanics of large elastic arteries (Lin et al., 2022), they are believed to play a more prominent structural role in muscular vessels (Leloup et al., 2015). Our Stage 1 calibration identifies a low elastin content, supporting that elastin plays a lesser role in the passive mechanics of the coronary microvasculature. This is reflected by *c*_1_ being the second least sensitive passive parameter. Stage 1 was generally sensitive to changes in both passive and active material parameters. In contrast, Stage 2 calibration was found to be relatively insensitive to perturbations in passive and active material parameters (Tables 1, 2, 3), with an average diameter change of <5% across the three homeostatic trees in response to 10% parameter perturbations. This difference in sensitivity is probably because Stage 1 is evaluated over a wide range of perfusion pressures, which captures the non‐linear mechanical response of the vessel wall. In contrast, the Stage 2 sensitivity function is evaluated at a single homeostatic perfusion pressure, where the material behaviour is more constrained.

Through our Stage 3 sensitivity analysis (Equation 24), we identified small arteriole pre‐stretches and $S_{max}$ as the most sensitive parameters of the model (Tables 1, 2, 3). This finding is physiologically reasonable, as small arteriole pre‐stretches directly influence stiffness of vessels that provide the greatest resistance to coronary flow, while $S_{max}$ plays a central role in determining the level of active tension.

By exploring six different combinations of autoregulatory mechanisms, we were able to investigate their relative contributions to coronary autoregulation (Fig. 11). We found that metabolic control was the most dominant in pressure–flow autoregulation, followed by myogenic, and then shear‐dependent (Fig. 11). Shear‐dependent control was found to only provide a minor contribution, and removing it only marginally impacted pressure–flow autoregulation, consistent with previous studies by Namani et al. (2018). The qualitative contributions between the three autoregulatory mechanisms agree with fig. 2A of Carlson et al. (2008) for arterioles supplying skeletal muscles. We found that switching between the Low and High $\phi_{myo}$ profile had a profound impact on the pressure–flow relationship, meaning the relative contribution of the myogenic response is strongly dependent on our normalized myogenic response distribution. Thus, this framework provides a means of investigating how longitudinal autoregulatory mechanisms contribute to global coronary autoregulation, which cannot be assessed experimentally. Additionally, by attenuating or removing individual control pathways, the framework can be used to simulate conditions that alter the responsiveness of coronary autoregulatory mechanisms (e.g. impaired myogenic tone or endothelial dysfunction).

Lastly, the utility of our microstructurally motivated framework for pathophysiological studies was demonstrated by examining how collagen stiffening or reduced SMC contractility affects coronary pressure–flow autoregulation. We found that collagen stiffening had minimal impact on pressure–flow autoregulation but significant impact on dilatory capacity (Fig. 12). This suggests that while adequate coronary flow may be maintained under resting conditions, the stiffer vessels exhibit insufficient vasodilatation during periods of increased demand. Halving $S_{max}$ resulted in complete loss of pressure–flow autoregulation, highlighting the critical role of SMC contractile function in maintaining coronary autoregulatory capacity. These examples demonstrate how the proposed framework can be used to investigate how vascular dysfunction impacts coronary autoregulation.

### Model limitations

The present study has several limitations. First, the experimental measurements delineating the pressure–diameter relationships of myocardial coronary vessels are scarce. We used four available sets of pressure–diameter data for a wide range of subepi, midwall and subendo vessels from 11 to 500 µm. More pressure–diameter data in the coronary arterioles will enhance the accuracy and predictive capabilities of the model. Second, coronary vascular networks were idealized to bifurcating trees with 12 generations, penetrating the myocardium using a simplified anatomical representation (see section A model for intramyocardial pressures). A more realistic reconstruction of the coronary network, potentially by utilizing the diameter‐defined Strahler order scheme (Kassab et al., 1993) or microvascular imaging (Sturgess et al., 2025), could impact local haemodynamics and autoregulatory responses. Third, the contribution of myocardial tethering is not considered in our model, which plays an important role in coronary vessel mechanics. Fourth, we use simplified models of the autoregulatory mechanisms. The mechanisms involved in the coronary autoregulation, especially the role of metabolic control, have been a largely debated issue in coronary physiology (Kiel et al., 2018). In this study we simulated coronary autoregulation using the three main mechanisms and their range of influence as defined in previous modelling studies. Our model, however, could be further refined with improvements in our understanding of the interplay between the different mechanisms of coronary autoregulation. Fifth, the framework uses continuum mechanics assumptions to model haemodynamics and vessel wall mechanics. In the smallest vessels in our coronary tree, discrete cellular and extracellular matrix organization may challenge these assumptions and may ultimately require formulations that explicitly represent structural discreteness (e.g. agent‐based models). However, we demonstrate that the CMM formulation can effectively capture the passive and active pressure–diameter relationship down to the small arterioles (Fig. 5*D*), suggesting that strain–energy functions still provide a useful framework for modelling wall mechanics at this scale. Furthermore, we use an *in vivo* viscosity law to model haemodynamics and account for non‐Newtonian blood rheology (Pries et al., 1994). The continuum formulations used in this study, combined with experimentally determined rheology laws, aim to provide an effective bulk viscosity for microvascular flow computations. However, velocity profiles and near wall shear stress lack the accuracy of formulations that explicitly account for interactions between blood constituents and plasma (Forouzandehmehr & Shamloo, 2018). Lastly, while our framework is calibrated using swine data, validation incorporates multiple species (e.g. the canine data in Fig. 10). Given the inherent interspecies variability in coronary physiology (Tune et al., 2020), the model would benefit from a more uniform approach utilizing calibration and validation datasets from a single species.

## Conclusion

The study of physiology and pathophysiology in coronary circulation benefits from accurate mathematical models that can account for both the microstructure and physiological function of arterial networks. In this study, we presented a microstructurally motivated coronary autoregulation model that uses a non‐linear continuum mechanics approach to account for the morphometry and vessel wall composition in three idealized coronary trees. Literature data were used to calibrate and test our model. With some modifications, this model can be applied to morphometry‐based coronary trees instead of idealized symmetric trees. Finally, since our model is based on constrained mixture theory, it could be expanded to also study long‐term growth and remodelling in the coronary circulation in response to hypertension, atherosclerosis, etc.

## Additional information

## Competing interests

The authors declare that they have no competing interests.

## Author contributions

M.J.E., H.G., S.B., D.A.B., J.D.T. and C.A.F. conceived the study design. M.J.E. and H.G. developed the model. All authors were involved in data analysis and interpretation. M.J.E., H.G. and C.A.F. prepared figures and drafted the manuscript. All authors reviewed and edited the manuscript. All authors approved the final version of the manuscript. All authors meet authorship criteria, acknowledge accountability for this work and confirm that no qualifying contributors have been omitted.

## Funding

This study was supported by the NIH R01‐HL158723, U01‐HL135842, and R01‐HL139813.

## Supporting information


Peer Review History

## Data Availability

Code and data supporting this study are available from the corresponding author upon reasonable request.

## Appendix A

### Mechanics of a single artery

A single vessel of the arterial tree is considered as a thin‐walled cylindrical tube composed of three main load‐bearing constituents: elastin (e), collagen (c) and SMCs. First, we only consider the passive response of constituents. Each constituent is assumed to separately contribute to the strain energy density:

(A1)
$$w=w^{e}\left(F_{n}^{e}\right)+w^{SMC}\left(F_{n}^{SMC}\right)+w^{c}\left(F_{n}^{c}\right),$$

where $F_{n}^{i}$ is the deformation gradient of each constituent i (i∈{e,SMC,c}) corresponding to its map from a stress‐free configuration to the overall homeostatic configuration. We define this deformation gradient as $F_{n}^{i}=FG^{i}$, where F represents the deformation gradient of the mixture mapping from its reference configuration to the homeostatic configuration, and $G^{i}$ is a pre‐stretch for each constituent mapping each constituent from its distinct stress‐free configuration to the reference configuration (Baek et al., 2005). In particular, the pre‐stretch mapping for elastin can be expressed as:

(A2)
$$G^{e}=\left(\begin{array}{ccc} G_{r}^{e} & 0 & 0\\ 0 & G_{\theta }^{e} & 0\\ 0 & 0 & G_{z}^{e} \end{array}\right)\;,$$

where $G_{\theta }^{e}$ and $G_{z}^{e}$ are pre‐stretches associated with circumferential and axial directions, and $G_{r}^{e}=1/G_{\theta }^{e}G_{z}^{e}$.

Similarly, for collagen fibres and SMCs, $\;M^{i}$, i∈{k,SMC} is defined as the unit vector in the direction of the collagen fibre (k) or SMCs. The pre‐stretch mappings for collagen and SMCs are given as:

(A3)
$$G^{k}=G_{h}^{c}\;M^{k}⊗M^{k},\;\;G^{\mathrm{SMC}}=G_{h}^{SMC}\;M^{\mathrm{SMC}}⊗M^{\mathrm{SMC}},\;$$

where the pre‐stretches $G_{h}^{c}$ and $G_{h}^{SMC}$ are also called homeostatic stretches, the stretches of the constituents when they are produced. Note that in the previous applications of growth and remodelling, the pre‐stretches were assumed to be constant for a single vessel. In our generalization of the framework to a vascular tree, we account for the variation of pre‐stretches across the generations of vessels. Nevertheless, the pre‐stretch implies that the homeostatic state in an individual vessel is associated with a constant homeostatic stress for the constituents of the vessel wall.

The orientation of collagen fibres and smooth muscles with respect to the axial direction in their reference configuration, defined by angle $\gamma^{k}$, can be written as:

(A4)
$$M^{k}=\cos\left(\gamma^{k}\right)\;e_{Z}+\sin\left(\gamma^{k}\right)e_{\theta },M^{m}=e_{\theta }.$$

For modelling the extension and inflation of a thin wall model, F is considered as $F\;=diag[\lambda_{r},\;\lambda_{\theta },\lambda_{z}].$ The stretches of constituent *i*, $\lambda^{i}$, i∈{e,k,SMC}, are expressed in terms of the pre‐stretches using $F^{i}=\;FG^{i}$:

(A5)
$$\begin{array}{ccc} & & \lambda_{\theta }^{e}=G_{\theta }^{e}\;\lambda_{\theta },\;\;\lambda_{z}^{e}=G_{z}^{e}\;\lambda_{z},\;\;\lambda^{k}=G_{h}^{c}\;\sqrt{\lambda_{\theta }^{2}sin^{2}\gamma^{k}+\lambda_{z}^{2}cos^{2}\gamma^{k}},\\ & & \lambda^{SMC}=G_{h}^{SMC}\;\lambda_{\theta }. \end{array}$$

The incompressibility of the wall material is imposed by assuming an isochoric motion [i.e. det(F)=1], and thus $\;\lambda_{r}=1/\lambda_{\theta }\lambda_{z}$. Using the membrane theory (Humphrey, 2002), the passive membrane Cauchy stress (force per deformed length) can be written as:

(A6)
$$T_{pas}=\frac{1}{J_{2D}}\;F\frac{\partial w}{\partial F^{T}}\Rightarrow \;T_{\theta \theta }=\frac{1}{\lambda_{z}}\;\frac{\partial w}{\partial \lambda_{\theta }},\;\mathrm{and}\;\;T_{zz}=\frac{1}{\lambda_{\theta }}\;\frac{\partial w}{\partial \lambda_{z}},\;$$

where $\;J_{2D}=\lambda_{\theta }\;\lambda_{z}$. The total strain energy per unit area can be written as:

(A7)
$$w=M_{R}^{e}\;\Psi^{e}+\sum_{k}M_{R}^{k}\Psi^{k}+M_{R}^{SMC}\Psi^{SMC},\;$$

where $M_{R}^{i}$, i∈{e,k,SMC} is the mass of each constituent per unit reference area. Alternatively, the total strain energy per unit area can be written as:

(A8)
$$w=M_{R}^{total}\;\left(\nu^{e}\Psi^{e}+\sum_{k}\nu^{k}\Psi^{k}+\nu^{SMC}\Psi^{SMC}\right),\;$$

where $\nu^{e}$, $\nu^{k}$ and $\nu^{SMC}$ are mass fractions of elastin, collagen fibre families and SMCs, respectively. In this work, four families of collagen fibres in circumferential, axial and two diagonal directions were considered with mass fractions $\nu^{k}=$(0.1, 0.1, 0.4, 0.4)$\nu^{c}$, where $\nu^{c}$ is the total collagen mass fraction (Zeinali‐Davarani et al., 2011). The total mass per unit area $M_{R}^{total}$ is the mass of load‐bearing constituents and can be computed via:

(A9)
$$M_{R}^{total}=\rho_{wall}\;H,$$

where $\rho_{wall}$ is the density of the vascular wall and H is the thickness under homeostatic conditions.

A neo‐Hookean model is employed for the passive elastin response and a Holzapfel exponential model is used for collagen fibre families and passive behaviour of circumferentially oriented SMCs:

(A10)
$$\Psi^{e}=\frac{c_{1}}{2}\;\left\{{\left(\lambda_{\theta }^{e}\right)}^{2}+{\left(\lambda_{z}^{e}\right)}^{2}+\frac{1}{{\left(\lambda_{\theta }^{e}\right)}^{2}{\left(\lambda_{z}^{e}\right)}^{2}}-3\right\}$$

(A11)
$$\Psi^{k}\;\left(\lambda_{n}^{k}\right)=\frac{c_{2}}{4c_{3}}\;\left\{\exp\left[c_{3}{\left(\lambda_{n}^{k^{2}}-1\right)}^{2}\right]-1\right\}$$

(A12)
$$\Psi^{SMC}\;\left(\lambda_{n}^{SMC}\right)=\frac{c_{4}}{4c_{5}}\;\left\{\exp\left[c_{5}{\left(\lambda_{n}^{SMC^{2}}-1\right)}^{2}\right]-1\right\},$$

where $c_{1}$ is the elastin material parameter, $c_{2}$ and $c_{3}$ are collagen material parameters, and $c_{4}$ and $c_{5}$ are passive SMC material parameters. To include the active tension of vascular SMCs, we use a potential function as given by (Baek et al., 2007):

(A13)
$$\Psi_{act}^{SMC}=\frac{S}{\rho_{wall}}\;\left\{\lambda_{\theta }+\frac{1}{3}\frac{{\left(\lambda_{M}-\tilde{\lambda }\right)}^{3}}{{\left(\lambda_{M}-\lambda_{0}\right)}^{2}}\right\},\;$$

where $\lambda_{M}$ and $\lambda_{0}$ are stretches at which the active force generation is maximum and zero, respectively, and $\rho_{wall}$ is the vascular wall density. In addition, $\tilde{\lambda }$ is an active stretch of the SMCs in the circumferential direction, which can evolve by SMC remodelling over slow timescales (hours to days). In the current study, we focus on the short timescale adaptations (minutes), and thus $\tilde{\lambda }$ is assumed to be the total circumferential stretch in the vessel ($\lambda_{\theta }).$ Active SMC stress S is in general a function of the myogenic, shear‐dependent and metabolic controls in coronary vessels. Again, using membrane theory, $\;T_{act}=\frac{1}{\lambda_{z}}\;\frac{\partial M_{R}^{SMC}\Psi_{act}^{SMC}}{\partial \lambda_{\theta }}$ the total tension in the artery can be written as:

(A14)
$$T=T_{pass}+T_{act}e_{\theta }⊗e_{\theta }.$$

Finally, for a thin‐walled cylinder with transmural pressure $p^{tv}$, the force equilibrium in the circumferential direction gives:

(A15)
$$\frac{p^{tv}D}{2}=T_{\theta \theta }+T_{act},$$

where D is the vessel diameter and $p^{tv}$ is the transvascular pressure of the artery.

We assume the reference configuration of the blood vessel in our continuum mechanics formulation is its homeostatic configuration. Therefore, by setting F=I (i.e. $\;\lambda_{\theta }=\lambda_{z}=1$) in equations above, we can write:

(A16)
$$\frac{p_{h}^{tv}D_{h}}{2}=T_{\theta \theta_{h}}+T_{act_{h}},\;$$

where $T_{\theta \theta_{h}}$ and $T_{act_{h}}$ are homeostatic passive and active circumferential tensions, respectively, $\;p_{h}^{tv}$ is the homeostatic transvascular pressure and $D_{h}$ is the homeostatic diameter of the vessel. The homeostatic passive tension $T_{\theta \theta_{h}}$ is thus determined by the material properties ($c_{1}$–$c_{5}$), constituent prestretches ($G_{\theta }^{e}$, $G_{z}^{e}$, $G_{h}^{c}$ and $G_{h}^{m}$) and their mass fractions $\nu^{i}$. In addition, the homeostatic active tension $T_{\theta \theta_{h}}$ is determined by active SMC parameters, $\lambda_{M},\;\lambda_{0}$ and $\;S_{h}$. Accordingly, we can also compute the homeostatic stress in each constituent $\sigma^{i}$ as a function of its material parameters, its homeostatic stretch $G^{i}$ and active SMC stress in case of SMCs.

## Appendix B

### Framework calibration

#### Stage 1 calibration: passive and myogenic response

Left anterior descending (LAD) and left circumflex (LCx) myography measurements from Liao and Kuo (1997) are used to determine the passive and myogenic response of vessels 180 µm (small arteries), 95 µm (large arterioles), 62 µm (intermediate arterioles) and 37 µm (small arterioles) in diameter. Identical myogenic responses are assumed for trees in the different myocardial depths (subepi, midwall and subendo) at $p^{tv}\leq$ 100 mmHg. This assumption is made given conflicting evidence regarding transmural differences in myogenic strength across the myocardium (Dick et al., 2018; Kuo et al., 1988; Sorop et al., 2008) within the experimental pressure range (15–100 mmHg).

To describe the passive response of the vessel, the active SMC contribution is removed from the strain energy function (i.e. $w_{act}^{SMC}=0$; see Equation 1). Conversely, the purely myogenic response is modelled by removing the metabolic and shear‐dependent stimuli in Equation (6), that is, $s_{meta}=\;s_{\tau }=0$, and considering an SMC stimulus $s_{h_{myo}}$ which neglects the metabolic and shear‐dependent contributions embedded in $s_{h}$. Thus, the total myogenic stimulus is defined as:

(B1)
$$s_{total_{myo}}=s_{myo}+s_{h_{myo}}.$$

Passive and myogenic response calibration is performed under the assumption that mechanical properties of constituents (material stiffnesses $c_{1}-c_{5}$ and active SMC parameters $S_{max}$, $\lambda_{M},$ $\lambda_{0}$) are constant across the vascular tree, but mass fractions, homeostatic deposition stretch of different constituents (pre‐stretches) and myogenic strength depend on vessel size and myocardial depth (Dick et al., 2018; Gharahi et al., 2023). Overall, identifying CMM parameters of 12 representative vessels (four vessels in each myocardial depth) requires estimating 80 parameters. Several physiological assumptions were made to avoid unrealistic values of model parameters. Constituent pre‐stretches <1 are not allowed in the model. Collagen fibres are assumed to have a relatively low pre‐stretch (1 < $G_{h}^{c}\;<$1.1), whereas elastin and SMC are allowed to have larger pre‐stretches ($G_{\theta }^{e}$, $G_{z}^{e}$ and $G_{h}^{m}$, respectively) (Valentín & Humphrey, 2009). These assumptions reflect the high turnover of collagen (leading to low pre‐stretch) compared to the much lower turnover of elastin and SMC. Furthermore, collagen fibre angles were prescribed to follow axial, circumferential and diagonal directions (e.g. 0°, 90° and 45°, respectively). Additionally, a homeostatic SMC activation ($A_{h}$) of 0.5 is targeted (Carlson et al., 2008), assuming an equal vasodilatory and vasoconstrictive capacity at the homeostatic state.

The error function for passive and myogenic calibration for a vessel is given by:

(B2)
$$\begin{array}{ccc} E_{1} & = & \sum_{p^{exp}}{\left(D_{pass}^{model}\left(p^{exp}\right)-D_{pass}^{exp}\left(p^{exp}\right)\right)}^{2}\\ & & +\;{\left(D_{myo}^{model}\left(p^{exp}\right)-D_{myo}^{exp}\left(p^{exp}\right)\right)}^{2}+{\left(A_{h}-0.5\right)}^{2}\;, \end{array}$$

where, $p^{exp}$ is the set of experimental pressures tested by Liao and Kuo (1997), and the different D terms refer to vessel diameters under different conditions: pass and myo indicate the purely passive and myogenic responses, respectively, while model and exp indicate model and experimental diameters, respectively. Liao and Kuo (1997) do not report a homeostatic pressure ($p_{h}$) or vessel thickness ($H_{h}$), both necessary for CMM parameter calibration. Therefore, we initially estimated them from the literature (Guo & Kassab, 2004) and iteratively updated them using the homeostatic trees determined in Stage 2 calibration.

Due to the large number of CMM parameters, Stage 1 calibration is performed iteratively. Parameters are classified as either global or local. Global parameters do not depend on vessel size or myocardial depth and include the material constants ($c_{1}-c_{5}$) and SMC active parameters $S_{max}$, $\lambda_{0}$ and $\lambda_{M}$. Note that SMC active material parameters are determined through literature values (Table 1). Local parameters are those which vary between the vessel sizes and include all pre‐stretches (G), mass fractions ($\upsilon^{e}$ and $\upsilon^{c}$) and myogenic strengths ($a_{myo}$ and $\phi_{myo}$). Note that SMC mass fraction is determined as: $\upsilon^{m}=1-\;\upsilon^{e}-\;\upsilon^{c}$.

The calibration procedure is performed by iteratively optimizing global and local parameters. For optimizing global parameters, local parameters are fixed, and global parameters are calibrated using Nelder–Mead optimization (Seyedsalehi et al., 2015). To calculate the global error, the local error of each individual vessel, as defined in Equation (B2), is summed. For optimizing local parameters, global parameters are fixed, and local parameters are calibrated over each individual vessel. We iterate between global and local parameter optimization until our parameters converge.

#### Stage 2 calibration: homeostatic coronary trees

The homeostatic morphometric (e.g. diameters), structural (e.g. thickness) and haemodynamic (e.g. wall shear stress) properties of the subtrees are determined using the homeostatic optimization model presented by Gharahi et al. (2023). The homeostatic values of two specific vessel properties, vessel diameter $D_{h}$ and SMC activation $A_{h}$, are obtained using cycle‐averaged haemodynamic quantities (i.e. the steady component in Womersley's solution).

Each subtree is assumed to be symmetrically bifurcating over 12 generations, with the first generation having a homeostatic diameter $D_{h}$ = 500 µm (Table B1). Constant inlet pressure and constant outflow boundary conditions are prescribed for each subtree (Table B1). Vessel lengths were determined as a function of vessel diameters using the morphometric data from Guo and Kassab (2004).

**Table B1:** Parameters for coronary homeostatic optimization

Parameter	Description	Unit	Value	Source
*N_gen_*	Number of generations	–	12	–
$D_{0}$	Diameter of first generation	μm	500	–
$p_{h}^{\mathrm{in}}$	Homeostatic coronary inlet pressure	mmHg	100	(Kassab et al., 1997)
$q_{\mathrm{subepi}}^{\mathrm{term}}$	Subepi homeostatic terminal flow	mm^3^/s	0.001375	(Namani et al., 2018; Sturgess et al., 2024)
$q_{\mathrm{midwall}}^{\mathrm{term}}$	Midwall homeostatic terminal flow	mm^3^/s	0.0015	(Namani et al., 2018)
$q_{\mathrm{subendo}}^{\mathrm{term}}$	Subendo homeostatic terminal flow	mm^3^/s	0.00165	(Namani et al., 2018; Sturgess et al., 2024)
$p_{\mathrm{subepi}}^{\mathrm{im}}$	Subepi mean intramyocardial pressure	mmHg	12.7	(Namani et al., 2018)
$p_{\mathrm{midwall}}^{\mathrm{im}}$	Midwall mean intramyocardial pressure	mmHg	27.6	(Namani et al., 2018)
$p_{\mathrm{subendo}}^{\mathrm{im}}$	Subendo mean intramyocardial pressure	mmHg	42.5	(Namani et al., 2018)
$\rho_{\mathrm{blood}}$	Blood density	kg/m^3^	1060	–

CMM parameters determined in Stage 1 calibration are assigned to individual vessels according to their homeostatic diameter $D_{h}$ (Small Arteries: $D_{h}$> 140 µm, Large Arterioles: 140 ≥ $D_{h}$> 80 µm, Intermediate Arterioles: 80 ≥ $D_{h}$> 50 µm, Small Arterioles: 50 ≥ $D_{h}$ µm) and myocardial depth. However, grouping vessels into four classes is crude, since considerable haemodynamic and morphological variability may exist within one vessel class. We have observed that when CMM parameters (specifically SMC tone) are kept constant across all classes, homeostatic optimization does not produce the experimentally measured thickness–diameter relationship. Therefore, we calibrate the homeostatic SMC activation parameter $A_{h}$ (Equation 11) such that each vessel attains its target thickness $H_{h}$. Appendix A outlines the mathematical relationship between $A_{h}$ and $H_{h}$, following mechanical equilibrium principles stated in Equations (3) and (4).

Using measured thickness–diameter relationships for LAD subepicardial vessels (Guo & Kassab, 2004), the following strategy is used to prescribe the target thickness $H_{h}$ for individual vessels. First, $A_{h}$ values in the subepi tree were adjusted, and the homeostatic hoop stress ($\sigma_{h}$) was obtained through: $\sigma_{h}=\frac{p_{h}^{tv}D_{h}}{2H_{h}}$. Then, $A_{h}$ in the midwall and subendo layers was adjusted to match the subepi hoop stress distribution, based on the hypothesis proposed by Choy and Kassab (2009) that hoop stress remains uniform across the myocardial wall for a given vessel size. This hypothesis is supported by experimental measurements showing transmural differences in coronary vessel wall thickness (e.g. thicker walls are found in the subepi region for vessels of similar diameters).

A key morphometric output of the homeostatic optimization is the bifurcation exponent ξ, which describes the diameter relationship between mother ($D_{m})$ and daughter ($D_{d1}$ and $D_{d2}$) vessels at each bifurcation as: $D_{m}^{\xi }=D_{d_{1}}^{\xi }+D_{d_{2}}^{\xi }$. Kaimovitz et al. (2008) reported ξ throughout the swine coronary circulation and found considerable heterogeneity, probably reflecting the size‐dependent functional roles of coronary vessels (Goodwill et al., 2017). In this work, we add a penalty term to the cost function used by Gharahi et al. (2023) to incorporate LAD ξ measurements by Kaimovitz et al. (2008). Details on the cost function formulation for homeostatic optimization are provided in Appendix D.

#### Stage 3 calibration: metabolic and shear‐dependent control

Calibration Stage 3 determines the metabolic $a_{meta}$ (Equation 8) and shear‐dependent $a_{\tau }$ (Equation 10) scaling coefficients using LAD pressure–flow autoregulation data in swine (Dick et al., 2018), while keeping the myogenic scaling coefficient $a_{myo}$ fixed. Nelder–Mead optimization is performed to determine $a_{\tau }$ and $a_{meta}$ such that the following error function is minimized:

(B3)
$$E_{3}=\sum_{j}\sum_{p^{exp}}{\left(\frac{q_{j}^{model}\left(p^{exp}\right)}{q_{h_{j}}^{model}}-\;\frac{q^{exp}\left(p^{exp}\right)}{q_{h}^{exp}}\right)}^{2}\;$$

where j∈ {subendo, midwall, subepi}, $p^{exp}$ is the set of experimental pressures tested (Dick et al., 2018), $q_{j}$ is the total flow to each coronary subtree j, and $q_{h_{j}}$ is the homeostatic flow going to coronary subtree j. The superscripts model and exp indicate the model and experimental flows, respectively. For a set of $a_{meta}$ and $a_{\tau }$, the regulated coronary flow ($q_{j}^{model}$) is calculated using the iterative procedure discussed in the section Framework algorithm: how to calculate an autoregulated state section and illustrated in Fig. 4.

## Appendix C

### Womersley solution admittance matrix

At a given non‐zero frequency ω, the Womersley's solution for transvascular pressure and flow are calculated as (Filonova et al., 2020; Mackenzie, 2021):

(C1)
$$P^{tv}\;\left(z,\omega \right)=P^{tv}\;\left(0,\omega \right)\;e^{\frac{-i\omega z}{c}}=\left(P\left(0,\omega \right)-P^{im}\left(0,\omega \right)\right)\;e^{\frac{-i\omega z}{c}}$$

(C2)
$$Q\;\left(z,\omega \right)=Q\left(0,\omega \right)\;e^{\frac{-i\omega z}{c}}=\frac{\pi D^{2}P^{\mathrm{tv}}\left(0,\omega \right)}{4c\rho_{\mathrm{fluid}}}\;\left(1-g\right)e^{\frac{-i\omega z}{c}}$$

where P(0,ω) is the inlet lumen pressure, $P^{im}\left(0,\omega \right)$ is the inlet intramyocardial pressure, $P^{tv}\left(0,\omega \right)$ is the inlet transvascular pressure and Q(0,ω) is the inlet flow, all in the frequency domain. The variable z is the longitudinal spatial coordinate, c is the pulse wave velocity, $\rho_{blood}$ is the blood density, $i=\sqrt{-1}$, and $g=2J_{1}\left(\wedge \right)/\wedge J_{0}\left(\wedge \right)$. $J_{0}$ and $J_{1}$ are Bessel functions of the first kind, and $\wedge =i^{\frac{3}{2}}\;\alpha_{Wom}$ with $\alpha_{Wom}$ being the Womersley number defined as $\alpha_{Wom}=R\sqrt{\omega \rho_{blood}/\mu }$ and μ being the blood viscosity. Utilizing Euler's formula, Equations (C1) and (C2) can be rewritten as:

(C3)
$$P^{tv}\;\left(z,\omega \right)=P^{tv}\;\left(0,\omega \right)\left(\cos\left(-\frac{\omega z}{c}\right)+isin\left(-\frac{\omega z}{c}\right)\right)$$

(C4)
$$\begin{array}{ccc} & & Q\;\left(z,\omega \right)=\frac{\pi D^{2}P^{tv}\left(0,\omega \right)}{4c\rho_{fluid}}\;\left(1-g\right)\\ & & \left(\cos\left(-\frac{\omega z}{c}\right)+isin\left(-\frac{\omega z}{c}\right)\right). \end{array}$$

For a vessel of length L, we wish to solve for the flow, provided pulsatile pressure boundary conditions. For notational simplicity, let $Q\left(0,\omega \right)\;\equiv q_{1}$, $Q\left(L,\omega \right)\;\equiv -q_{2}$, $P^{tv}\left(0,\omega \right)\;\equiv \;p_{1}^{tv}$ and $P^{tv}\left(L,\omega \right)\;\equiv \;p_{2}^{tv}$. We seek an expression of the form: $q\;=\;Yp^{\mathrm{tv}}$, where Y is an admittance matrix, $q\;={\left[q_{1}\;q_{2}\right]}^{T}$ and $p^{\mathrm{tv}}={\left[p_{1}^{tv}\;p_{2}^{tv}\right]}^{T}$. To solve for $p^{\mathrm{tv}}$ and q, we plug z=0 and z=L into Equations (C3) and (C4). This results in:

(C5)
$$p_{1}^{tv}=P^{tv}\;,$$

(C6)
$$p_{2}^{tv}=P^{tv}\;\nu ,$$

(C7)
$$q_{1}=mP^{tv},$$

(C8)
$$q_{2}=-mP^{tv}\nu ,$$

with $\nu \;\equiv \;C_{L}+iS_{L}$, $m\equiv \;\frac{\pi D^{2}}{4c\rho_{fluid}}\left(1-g\right)$, $S_{L}\equiv sin\left(-\frac{\omega L}{c}\right)$ and $C_{L}\equiv cos\left(-\frac{\omega L}{c}\right)$. Equations (C5)–(C8) are written as a system of equations:

(C9)
$$Y_{11}+Y_{12}\nu =m,$$

(C10)
$$Y_{21}+Y_{22}\nu =-m\nu .$$

Furthermore, we have $Y_{11}=Y_{22}$ and $Y_{12}=Y_{21}$ due to the symmetric nature of the system. This results in the admittance matrix:

(C11)
$$Y=\frac{m}{1-\nu^{2}}\left[\begin{array}{cc} 1+v^{2} & -2v\\ -2v & 1+v^{2} \end{array}\right].$$

## Appendix D

### Homeostatic optimization

Originally introduced by Murray (1926), and later extended by Taber (1998) and Lindström et al. (2015), the extended Murray's law states that the vessel wall composition and geometry strive to minimize the energy consumption. The homeostatic optimization framework proposed in Gharahi et al. (2023) utilizes this idea to identify the homeostatic characteristics of an arterial tree. Briefly, the total energy cost per unit length C for an individual blood vessel can be written as:

(D1)
$$\begin{array}{ccc} & & C\;\left(M_{R}^{i},R;q\right)=C_{blood}+C_{drag}+C_{wall}\\ & & =\;\frac{\alpha^{blood}\pi D^{2}}{4}+\frac{128\mu q^{2}}{\rho_{blood}D^{4}}+\frac{\pi D}{\rho_{wall}}\sum_{i}\alpha^{i}M_{R}^{i}, \end{array}$$

where $C_{blood}$ is the metabolic cost of sustaining blood in the vessels, $C_{drag}$ is the power needed to overcome viscous drag and $C_{wall}$ is the metabolic cost of maintaining the constituents of the wall. In addition, $\alpha^{blood}$ and $\alpha^{i}$ are the metabolic energy costs of blood and wall constituents (i∈{e,c,SMC}, see Appendix A) per unit volume, q is the blood flow in the vessel, and μ, $\rho_{blood}$ and $\rho_{wall}$ are the blood viscosity, blood density and vessel wall density, respectively. Note that the metabolic cost of SMCs can be written as the summation of the metabolic cost of their maintenance $\alpha_{maint}^{m}$ and the metabolic cost of the active SMC stress $\alpha_{active}^{m}$.

(D2)
$$\alpha^{m}=\alpha_{maint}^{m}+\alpha_{active}^{m}S_{h},$$

where $S_{h}$ is the homeostatic active SMC stress. This metabolic cost function must be minimized with Equation (A16) as the constraint in each segment. With regard to Appendix A, we can rewrite the mass of constituents in terms of their mass fractions as $\nu^{e}=M_{R}^{e}\;/M_{R}^{total}$, $\nu^{m}=M^{m}\;/M_{R}^{total}$ and $\nu^{k}=M_{R}^{k}\;/M_{R}^{total}$ with $\nu^{c}=\Sigma \nu^{k}$. Using the mass fractions and considering that the arteries are in homeostatic state, we can write the mechanical equilibrium in each vessel as

(D3)
$$\frac{p^{tv}D}{2}=\frac{M_{R}^{total}}{\rho_{wall}}\;\sum_{i}\nu^{i}\sigma_{\theta }^{i},$$

where $\sigma_{\theta }^{i}$ is the circumferentially acting part of the homeostatic stress tensor $\sigma^{i}$. Therefore, the energy cost of one vessel can be written in terms of diameter, with pressure and flow rate given from the haemodynamics as

(D4)
$$C\;\left(R;q,p_{tm}\right)=\frac{\alpha^{blood}\pi D^{2}}{4}+\frac{128\mu q^{2}}{\rho_{blood}D^{4}}+\frac{\pi p^{tv}D^{2}}{2}\frac{\sum_{i}\nu^{i}\alpha^{i}}{\sum_{i}\nu^{i}\sigma_{\theta }^{i}},$$

The total energy cost of all arteries can be added to compute the total cost for maintenance of an arterial tree

(D5)
$$C_{total}=\sum_{j}C_{j}L_{j}\;N_{j},$$

where $C_{j}$ is the energy cost (Equation D4), $L_{j}$ is the length of vessels in generation j and $N_{j}$ is the number of vessels in generation j. In this study, we add the bifurcation diameter exponent (ξ) measurement from Kaimovitz et al. (2008) to the cost function.

(D6)
$$C_{total}=\sum_{j}C_{j}L_{j}N_{j}+k{\left(\xi_{model}-\xi_{data}\right)}^{2},$$

where $\xi_{model}$ is the bifurcation diameter exponent in the tree, $\xi_{data}$ is the measured bifurcation diameter exponent and k is a penalty coefficient. Once the radius of each vessel is determined by minimization of $C_{total}$, the thickness of each vessel can be computed. As explained in Appendix A, the homeostatic stress tensor $\sigma^{i}$ for each constituent is a function of its material parameters, its homeostatic pre‐stretches and the active SMC stress. In this work, to find the homeostatic characteristics of the vessel wall in the tree, material parameters and pre‐stretches are determined according to their diameter using the four types of vessels: small arteries, large, intermediate and small arterioles. However, the SMC active stress S is tuned for each individual vessel in the tree by changing SMC activation A to fit the thickness to diameter ratio reported in Guo and Kassab (2004). The parameters used for the homeostatic optimization are summarized in Table D1..

**Table D1:** Parameters used in homeostatic optimization

Parameter	Description	Value	Unit	Reference
$\alpha^{c}$ and $\alpha_{maint}^{m}$	Metabolic cost of collagen and SMCs per unit volume	1070	W/m^3^	(Paul, 2011)
$\alpha^{e}$	Metabolic cost of elastin per unit volume	0 (Only produced at early ages.)	W/m^3^	(Lindström et al., 2015)
$\alpha^{blood}$	Metabolic cost of blood per unit volume	51.7	W/m^3^	(Liu et al., 2012)
$\alpha_{active}^{m}$	Metabolic cost of active SMC stress	0.00872	1/s	(Paul, 2011)
$\rho_{wall}$, $\rho_{blood}$	Vessel and blood density	1060	kg/m^3^	–
