# Supplementary material for: A microstructurally motivated framework to study autoregulation in the coronary circulation
Source: J Physiol. 2026 Jun 16;604(14):5892–925. doi: 10.1113/JP291222 (PMC13370741; doi:10.1113/JP291222)
Supplement: Supplementary file 1 — Peer Review History [file TJP-604-5892-s001.pdf]

The Journal of Physiology

A Microstructurally-Motivated Framework to Study Autoregulation in the Coronary Circulation

Matthew J Eden, Hamid Gharahi, Victoria E Sturgess, Domingo E Uceda, Greg Dick, Seungik Baek, Daniel A Beard, Johnathan D. Tune, and C. Alberto Figueroa
DOI: 10.1113/JP291222

Corresponding author(s): C. Alberto Figueroa (figueroc@med.umich.edu)

The following individual(s) involved in review of this submission have agreed to reveal their identity: Amin Forouzandehmehr (Referee #1)

Review Timeline:

Submission Date:	19-Feb-2026
Editorial Decision:	18-Mar-2026
Revision Received:	06-May-2026
Accepted:	15-May-2026

Senior Editor: Natalia Trayanova

Reviewing Editor: Diego Candia-Rivera

Transaction Report:

(Note: With the exception of the correction of typographical or spelling errors that could be a source of ambiguity, letters and reports are not edited. Depending on transfer agreements, referee reports obtained elsewhere may or may not be included in this compilation. Referee reports are anonymous unless the Referee chooses to sign their reports.)

Re: JP-RP-2026-291222 "**A Microstructurally-Motivated Framework to Study Autoregulation in the Coronary Circulation**" by Matthew J Eden, Hamid Gharahi, Victoria E Sturgess, Domingo E Uceda, Greg Dick, Seungik Baek, Daniel A Beard, Johnathan D. Tune, and Alberto Figueroa

Dear Dr Figueroa,

Thank you for submitting your manuscript to The Journal of Physiology. It has been assessed by a Reviewing Editor and by 2 expert referees and we are pleased to tell you that it is potentially acceptable for publication following satisfactory major revision.

Please advise your co-authors of this decision as soon as possible.

The referee reports are copied at the end of this email.

Please address all the points raised and incorporate all requested revisions or explain in your Response to Referees why a change has not been made. We hope you will find the comments helpful and that you will be able to return your revised manuscript within 2 months. If your article is NOT for a Special Issue, you may have 9 months to revise. If you require an extension, please contact journal staff: jp@physoc.org. Please note that this letter does not constitute a guarantee for acceptance of your revised manuscript.

Your revised manuscript should be submitted online using the link in your Author Tasks: Link Not Available. This link is accessible via your account as Corresponding Author; it is not available to your co-authors. If this presents a problem, please contact journal staff (jp@physoc.org). Image files from the previous version are retained on the system. Please ensure you replace or remove any files that are being revised.

If you do not wish to submit a revised version of your manuscript, you must inform our journal staff (jp@physoc.org) or reply to this email to request withdrawal. Please note that a manuscript must be formally withdrawn from the peer review process at one journal before it may be submitted to another journal.

TRANSPARENT PEER REVIEW POLICY: To improve the transparency of its peer review process, The Journal of Physiology publishes online as supporting information the peer review history of all articles accepted for publication. Readers will have access to decision letters, including Editors' comments and referee reports, for each version of the manuscript, as well as any author responses to peer review comments. Referees can decide whether or not they wish to be named on the peer review history document.

ABSTRACT FIGURES: Authors are expected to use The Journal's premium BioRender account to create/redraw their Abstract Figures. Information on how to access this account is here: https://physoc.onlinelibrary.wiley.com/journal/14697793/biorender-access.

This will enable Authors to create and download high-resolution figures. If authors have used the free BioRender service, they can use the instructions provided in the link above to download a high-resolution version suitable for publication.

The link provided should only be used for the purposes of this submission. Authors will be charged for figures created on this account if they are not related to this manuscript submission.

LANGUAGE EDITING AND SUPPORT FOR PUBLICATION: If you would like help with English language editing, or other article preparation support, Wiley Editing Services offers expert help, including English Language Editing, as well as translation, manuscript formatting, and figure formatting at www.wileyauthors.com/eeo/preparation. You can also find resources for Preparing Your Article for general guidance about writing and preparing your manuscript at www.wileyauthors.com/eeo/prepresources.

RIGOUR AND REPRODUCIBILITY: We recommend authors consult The Journal's Rigour and Reproducibility requirements to ensure that their manuscript is suitable for publication: https://physoc.onlinelibrary.wiley.com/pb-assets/hub-assets/physoc/documents/TJP-Rigour-and-Reproducibility-Requirements.pdf

REVISION CHECKLIST:

Check that your Methods section conforms to journal policy: https://jp.msubmit.net/cgi-bin/main.plex?form_type=display_requirements#methods.

Check that data presented conforms to the statistics policy: https://jp.msubmit.net/cgi-bin/main.plex?form_type=display_requirements#statistics.

Upload a full Response to Referees file. To create your 'Response to Referees': copy all the reports, including any comments from the Senior and Reviewing Editors, into a Microsoft Word, or similar, file and respond to each point, using font or background colour to distinguish comments and responses and upload as the required file type.

Please upload two versions of your manuscript text: one with all relevant changes highlighted and one clean version with no changes tracked. The manuscript file should include all tables and figure legends, but each figure/graph should be uploaded as separate, high-resolution files.

You may also upload:

- 'Potential Cover Art' for consideration as the issue's cover image
- Appropriate Supporting Information (Video, audio or data set: see https://jp.msubmit.net/cgi-bin/main.plex?form_type=display_requirements#supp).

We look forward to receiving your revised submission.

If you have any queries, please reply to this email and we will be pleased to advise.

Yours sincerely,

Natalia Trayanova
Senior Editor
The Journal of Physiology

REQUIRED ITEMS

- Author photo and profile. First or joint first authors are asked to provide a short biography (no more than 100 words for one author or 150 words in total for joint first authors) and a portrait photograph. These should be uploaded and clearly labelled together in a Word document with the revised version of the manuscript. See Information for Authors for further details.

- The contact information for the person responsible for 'Research Governance' at your institution needs to be provided. This includes their name and an institutional email address. Please ensure the contact is not an author on this paper and provide an alternate contact if necessary, or confirm in the submission form that the author whose email was provided has sole responsibility for research governance. This is the person who is responsible for regulations, principles and standards of good practice in research carried out at the institution, for instance the ethical treatment of animals, the keeping of proper experimental records or the reporting of results.

- The reference list must be in alphabetical order, rather than numbered, to comply with our Journal format.

- Your manuscript must include a complete Additional Information section, including competing interests; funding; author contributions and acknowledgements.

- Please upload separate high-quality figure files via the submission form.

- Please ensure that any tables are editable and in Word format, and wherever possible, embedded in the article file itself.

- Please ensure that the Article File you upload is a Word file.

- Papers must comply with the Statistics Policy: https://jp.msubmit.net/cgi-bin/main.plex?form_type=display_requirements#statistics.

In summary:

- If n {less than or equal to} 30, all data points must be plotted in the figure in a way that reveals their range and distribution.

A bar graph with data points overlaid, a box and whisker plot or a violin plot (preferably with data points included) are acceptable formats.

- If n > 30, then the entire raw dataset must be made available either as supporting information, or hosted on a not-for-profit repository, e.g. FigShare, with access details provided in the manuscript.

- 'n' clearly defined (e.g. x cells from y slices in z animals) in the Methods. Authors should be mindful of pseudoreplication.

- All relevant 'n' values must be clearly stated in the main text, figures and tables.

- The most appropriate summary statistic (e.g. mean or median and standard deviation) must be used. Standard Error of the Mean (SEM) alone is not permitted.

- Exact p values must be stated. Authors must not use 'greater than' or 'less than'. Exact p values must be stated to three significant figures even when 'no statistical significance' is claimed.

- A Data Availability Statement is required for all papers reporting original data. This must be in the Additional Information section of the manuscript itself. It must have the paragraph heading 'Data Availability Statement'. All data supporting the results in the paper must be either: in the paper itself; uploaded as Supporting Information for Online Publication; or archived in an appropriate public repository. The statement needs to describe the availability or the absence of shared data. Authors must include in their statement: a link to the repository they have used, or a statement that it is available as Supporting Information; reference the data in the appropriate sections(s) of their manuscript; and cite the data they have shared in the References section. Whenever possible, the scripts and other artefacts used to generate the analyses presented in the paper should also be publicly archived. If sharing data compromises ethical standards or legal requirements then authors are not expected to share it, but must note this in their statement. For more information, see our Statistics Policy.

- Please include an Abstract Figure file and an Abstract Figure legend. An appropriate figure legend, which should not exceed 150 words in length, should be included in the main manuscript file. The Abstract Figure is a piece of artwork designed to give readers an immediate understanding of the research and should summarise the main conclusions. If possible, the image should be easily 'readable' from left to right or top to bottom. It should show the physiological relevance of the manuscript so readers can assess the importance and content of its findings. Abstract Figures should not merely recapitulate other figures in the manuscript. Please try to keep the diagram as simple as possible and without superfluous information that may distract from the main conclusion(s). Abstract Figures must be provided by authors no later than the revised manuscript stage and should be uploaded as a separate file during online submission labelled as File Type 'Abstract Figure'. Please also ensure that you include the figure legend in the main article file. All Abstract Figures should be created using BioRender. Authors should use The Journal's premium BioRender account to export high-resolution images. Details on how to use and access the premium account are included as part of this email.

EDITOR COMMENTS

The manuscript has now been reviewed by two expert referees and myself. The work presents an original mechanistic framework for coronary autoregulation. However, certain limitations arise from the simplifications and the apparently arbitrary selection of parameters and model constraints. In addition, further clarification is needed regarding the translational potential of this development.

In view of the referees' comments, your manuscript is not suitable for publication in its current form. Please consider submitting a revised version for further evaluation.

The manuscript presents a re-analysis of previously acquired and published data. Please state this explicitly in the Methods section and include details of the associated ethics approval from the original studies.

REFEREE COMMENTS

Referee #1:

This manuscript presents a sophisticated and timely computational framework for coronary autoregulation that integrates microstructurally motivated vessel wall mechanics, constrained mixture theory, and network-scale hemodynamics. The work

is ambitious in scope and offers a mechanistically rich platform capable of linking short-term tone regulation with long-term vascular remodeling. The framework is calibrated to literature data and addresses important heterogeneities across myocardial layers. Overall, the study is technically strong and potentially impactful for computational physiology and coronary pathophysiology research. However, several methodological assumptions require clearer justification, and some aspects of model generalizability and physical consistency merit deeper discussion. I believe addressing the points below would substantially strengthen the manuscript.

Majors:

1. The authors appropriately emphasize transmural heterogeneity, yet an important limitation that should be explicitly acknowledged is whether the modeled trees correspond to the LAD, LCx, or RCA territories. Anatomical, morphometric, and flow characteristics differ substantially across these beds. The dependence of autoregulatory behavior on coronary territory could influence both calibration and interpretation of transmural differences.

2. The manuscript should explicitly report the diameter range represented across the vascular tree generations. A substantial portion of the model appears to involve vessels below ~100 µm, where discrete RBC effects become significant and continuum assumptions break down. Prior studies show that neglecting particulate blood effects in this regime can introduce nontrivial errors in viscosity, velocity profiles, and wall shear stress (e.g., https://doi.org/10.1007/s10237-017-0955-x). The authors should justify the continuum framework in this diameter range or discuss expected error bounds.

3. A related concern applies to vessel wall modeling. Deformation gradients and strain-energy formulations are continuum constructs. For microvessels approaching cellular length scales, structural discreteness of SMCs and ECM may invalidate homogenized assumptions. A discussion on the scale limits of constrained mixture theory would be valuable.

4. The manuscript employs Womersley's framework with a viscosity law, but does not justify the choice relative to hematocrit-dependent non-Newtonian formulations (e.g., Carreau-Yasuda models incorporating Fåhræus-Lindqvist effects). The authors should explain why a more explicitly hematocrit-sensitive rheology was not used and discuss how this choice might affect shear stress and flow waveforms.

5. Sensitivity analysis focuses on the autoregulatory error function. It would be informative to also assess sensitivity of key physiological outputs (e.g., transmural flow ratio, diameter changes, shear stress distribution) to better contextualize parameter importance.

6. The framework is positioned for pathophysiological studies, yet no disease case is demonstrated. Including at least one illustrative pathological scenario (e.g., impaired myogenic tone, collagen stiffening, endothelial dysfunction) would enhance the impact.

Minors:

1. There are several image-based computational studies on coronary fluid-solid interaction and network biomechanics. Including a concise review of these works in the Introduction or Discussion would better contextualize the contribution.

2. If "wall stress" refers to wall shear stress, terminology should be unified throughout the manuscript to avoid confusion with circumferential or hoop stress.

3. "In additional to" should be corrected to "In addition to".

4. A brief rationale for selecting 12 generations would help readers understand anatomical completeness.

5. A clear ranking or sensitivity-based decomposition of passive element contributions (e.g., elastin vs collagen vs passive SMC) would help interpret whether predicted vessel behavior aligns with known biomechanical literature.

Referee #2:

The manuscript by Eden et al. presents - to the best of my knowledge - a novel computational framework for coronary pressure-flow autoregulation based on constrained mixture models.

A three-stage calibration uses swine myography data for passive and myogenic vessel wall properties, Murray's law optimization for homeostatic morphometry, and swine pressure-flow data for metabolic/shear-dependent scaling. An extended Womersley solution accounts for pulsatile hemodynamics under time-varying intramyocardial pressure.

This is a very comprehensive contribution that is novel and interesting to the readers of J Phys.

Regarding impact and originality, to the best of my knowledge this is the first CMT-based coronary autoregulation framework, and its multi-scale capability is an advance over existing models.

The model provides mechanistic insight into transmural autoregulatory heterogeneity and the dominance of metabolic control.

Regarding study design and validity, calibration and validation datasets are largely independent, though all calibration data derive from swine while comparisons are mostly to canine data, as mentioned below in the specific concerns.

All data assessed in this manuscript are retrospective/previously published. I have no ethical concerns regarding this submission.

Overall, I recommend a revision addressing the following (minor) issues:

1) Inter-species calibration while comparisons (e.g. Fig 10) were mostly made to canine data. There is limited data available for calibration and validation, but the authors should

acknowledge this in the limitations section.

In that regard also note that in l801: "[...] wall shear stress tends to be greater than that measured by Stepp et al. (1999) in canines [32], but agrees better with previous modeling work by Cornelissen et al. (2002) [11]." Cornelissen et al. appears to be based on porcine data, which should be noted when drawing this comparison.

2) Sensitivity analysis seems to be only assessed for stage 3? What about stage 1 and 2? Also the diameter exponent \xi seems to be a bit above the values reported by Kaimovitz et al. 2008. What would be the impact of this on downstream hemodynamics?

3) Additional papers worth looking at could be: 1. Namani et al. 2020, DOI: 10.1152/ajpheart.00563.2019, overview of coronary autoregulation modeling; 2. Hoffman & Spaan (1990), DOI: 10.1152/physrev.1990.70.2.331, potentially in Section 4.4; or Wu et al. (2021), DOI: 10.1016/j.jmbbm.2020.104161, for determining constituent pre-stretches in CMT vascular models.

END OF COMMENTS

EDITOR COMMENTS

The manuscript has now been reviewed by two expert referees and myself. The work presents an original mechanistic framework for coronary autoregulation. However, certain limitations arise from the simplifications and the apparently arbitrary selection of parameters and model constraints. In addition, further clarification is needed regarding the translational potential of this development.

In view of the referees' comments, your manuscript is not suitable for publication in its current form. Please consider submitting a revised version for further evaluation.

The manuscript presents a re-analysis of previously acquired and published data. Please state this explicitly in the Methods section and include details of the associated ethics approval from the original studies.

Response: The authors thank Dr. Trayanova for coordinating the review process and for her thoughtful evaluation of our manuscript. We have revised the text to address the expert referees' comments, with detailed point-by-point responses provided below. Response to the reviewer's comments is given in red roman fonts. Text added to the manuscript is in *red italics fonts*.

An Ethical Statement (Section 2.8) has been added to explicitly state that this study represents a secondary analysis of previously published data. Furthermore, we have detailed the specific sources utilized for model development and calibration, along with their associated ethical approvals.

During the revision the authors identified a sign error when plotting the autoregulatory signals in Figure 9D-F and K-M, which has been corrected. This plotting error does not impact any additional results or conclusions from the manuscript, and the text has been updated to reflect the sign change, isolated to Section 3.3. The authors sincerely apologize for this oversight.

REFEREE COMMENTS
Referee #1
This manuscript presents a sophisticated and timely computational framework for coronary autoregulation that integrates microstructurally motivated vessel wall mechanics, constrained mixture theory, and network-scale hemodynamics. The work is ambitious in scope and offers a mechanistically rich platform capable of linking short-term tone regulation with long-term vascular remodeling. The framework is calibrated to literature data and addresses important heterogeneities across myocardial layers. Overall, the study is technically strong and potentially impactful for computational physiology and coronary pathophysiology research. However, several methodological assumptions require clearer justification, and some aspects of model generalizability and physical consistency merit deeper discussion. I believe addressing the points below would substantially strengthen the manuscript.

Response: We thank Referee #1 for their thoughtful and constructive feedback of the manuscript, and for identifying key areas where the manuscript can be strengthened. Our point-by-point response to their comments are provided below.

Majors:

Comment 1: The authors appropriately emphasize transmural heterogeneity, yet an important limitation that should be explicitly acknowledged is whether the modeled trees correspond to the LAD, LCx, or RCA territories. Anatomical, morphometric, and flow characteristics differ substantially across these beds. The dependence of autoregulatory behavior on coronary territory could influence both calibration and interpretation of transmural differences.

Response: Based on the sources of most references used in this work, the modeled trees most closely reflect the LAD microvascular territory. However, some literature data used for calibration and validation are drawn from studies reporting measurements in the LCx and/or more generally the left coronary artery (LCA) territories, as summarized in the table below. We agree with the reviewer that it is important to specify the anatomical territory calibration and validation data comes from. We now state the coronary territory associated with each literature dataset used throughout the manuscript. Additionally, we note at the beginning of Section 2.6 that the modeled trees should be interpreted as most representative of an LAD territory.

Publications used for model calibration and validation, with coronary territories.

Reference	Measurement (Figure)	Use in manuscript	Coronary territory
(Liao & Kuo, 1997)	○ Passive and myogenic diameter vs. pressure (Fig. 5A-D)	Stage 1 calibration	LAD & LCx
(Kaimovitz *et al.*, 2008)	○ Diameter-exponent vs. diameter (Fig. 6B)	Stage 2 calibration	LAD
(Guo & Kassab, 2004)	○ Subepi thickness vs. diameter (Fig. 6D)<br>○ Midwall and subendo hoop stress vs. diameter (Fig. 6F)	Stage 2 calibration	LAD
(Kassab *et al.*, 1997)	○ Lumen pressure vs. diameter (Fig. 6G)<br>○ Flow vs. diameter (Fig. 6H)	Stage 2 validation	LCA
(Stepp *et al.*, 1999)	○ Wall shear stress vs. diameter (Fig. 6I)	Stage 2 validation	LCA

(Dick *et al.*, 2018)	○ Autoregulated flow vs. perfusion pressure (Fig. 7A)	Stage 3 calibration	LAD
(Boatwright *et al.*, 1980)	○ ENDO/EPI vs. perfusion pressure (Fig. 10A)	Model validation	LCA
(Kanatsuka *et al.*, 1990)	○ ENDO/EPI vs. perfusion pressure (Fig. 10A)	Model validation	LAD
(Canty, 1988)	○ ENDO/EPI vs. perfusion pressure (Fig. 10A)	Model validation	LCA
(Kanatsuka *et al.*, 1990)	○ ΔD vs. diameter (Fig. 10B-C)	Model validation	LCA

Comment 2: The manuscript should explicitly report the diameter range represented across the vascular tree generations. A substantial portion of the model appears to involve vessels below ~100 µm, where discrete RBC effects become significant and continuum assumptions break down. Prior studies show that neglecting particulate blood effects in this regime can introduce nontrivial errors in viscosity, velocity profiles, and wall shear stress (e.g., https://doi.org/10.1007/s10237-017-0955-x). The authors should justify the continuum framework in this diameter range or discuss expected error bounds.

Response: The reviewer raises an important point. Indeed, a substantial portion of the model involves vessels below 100 µm, where continuum assumptions break down due to complex RBC-plasma interactions.

The relationship between vessel diameter and tree generation is reported in Figure 6A. We have also updated Section 3.1.2 and the Figure 6 caption to hopefully clarify this result. Regarding the complex interactions between RBC and plasma, our framework adopted the experimentally-determined non-Newtonian blood rheology model of Pries *et al.*, (1994), which was fitted with data in the 1000-3 µm vessel diameter range. We have added paragraphs discussing this important point in Section 2.4 (Hemodynamic modeling) citing the provided reference, and Section 4.6 (Model limitations).

Comment 3: A related concern applies to vessel wall modeling. Deformation gradients and strain-energy formulations are continuum constructs. For micro vessels approaching cellular length scales, structural discreteness of SMCs and ECM may invalidate homogenized assumptions. A discussion on the scale limits of constrained mixture theory would be valuable.

Response: We agree that as vessel diameters approach cellular length scales, the assumption of a homogenized continuum becomes strained due to the structural discreteness of SMCs and ECM components. Ultimately, formulations that explicitly represent structural discreteness (e.g., agent-based models) may be needed if the focus is to study processes at that scale. However, the emphasis of our work is in describing accurate pressure-diameter behavior while accounting for the heterogenous composition of the wall constituents. The strain-energy formulation provides a convenient mathematical framework, and has been applied at similar length scales in the pulmonary microcirculation (Gharahi *et al.*, 2023; Szafron *et al.*, 2023).

Following the reviewer's suggestion, we have updated Section 4.6 (Model limitations) to state that the assumptions of our continuum framework are stretched at the small length scales our model covers.

<u>Comment 4:</u> The manuscript employs Womersley's framework with a viscosity law, but does not justify the choice relative to hematocrit-dependent non-Newtonian formulations (e.g., Carreau-Yasuda models incorporating Fåhræus-Lindqvist effects). The authors should explain why a more explicitly hematocrit-sensitive rheology was not used and discuss how this choice might affect shear stress and flow waveforms.

Response: As described in our answer to Comment 2, the framework uses the in-vivo non-Newtonian viscosity law derived by (Pries *et al.*, 1994), which considers vessels ranging from 1000-3 μm in diameter and accounts for Fåhræus-Lindqvist effects as a function of blood hematocrit levels. The formulation was originally provided in Appendix D; however, to improve clarity we have removed Appendix D and added the viscosity law formulation in Section 2.4.1.

<u>Comment 5:</u> Sensitivity analysis focuses on the autoregulatory error function. It would be informative to also assess sensitivity of key physiological outputs (e.g., transmural flow ratio, diameter changes, shear stress distribution) to better contextualize parameter importance.

Response: We thank the reviewer for this suggestion. As requested by the reviewer, we expanded our sensitivity studies to focus on transmural flow ratios and diameter changes, using the analyses presented in Figures 11 (influence of autoregulatory mechanisms) and 12 (passive and active mechanics). As a result of these additional sensitivity studies, the updated Figures 11 and 12 now depict the sensitivity of ENDO/EPI ratios as a function of perfusion pressure, as well as changes in vessel diameters in response to mild and severe decreases in perfusion pressure. Given the length of the manuscript, and the extensive number of model parameters and physiological outputs, we have focused our expanded sensitivity analysis on areas most critical for model interpretation. This implies that due to the small contribution of shear-dependent control towards autoregulation (Figure 11), we have elected not to perform a dedicated sensitivity analysis on how changes in model parameters impact the wall shear stress distribution.

Furthermore, in response to reviewer #2, we further expanded our sensitivity analyses by calculating two new sensitivity metrics (X_1 and X_2), which quantify the impact of model parameters on Stage 1 and Stage 2 of model calibration. The X_1 sensitivity metric quantifies changes in the error function used to calibrate vessel wall mechanics (Stage 1) in response to a 10% change in individual parameters. The X_2 sensitivity metric quantifies the average change in homeostatic vessel diameter across the tree generations in response to a 10% change in individual parameters. X_1 and X_2 have been incorporated into all tables in the body of the manuscript (see below). To accommodate this additional information while maintaining readability, the original Table 1 was divided into two separate tables (Tables 1 and 2).

Lastly, regarding the contextualization of the importance of model parameters, we found Stage 1 to be more sensitive to changes in passive and active material properties, when compared to Stage 2 and 3 (see Tables 1-3). Several model parameters which were found to be particularly sensitive included: maximum SMC stress $\boldsymbol{S_{max}}$, Maximum active tension stretch $\boldsymbol{\lambda_M}$, and collagen pre-stretch $\boldsymbol{G_h^c}$ (particularly in the small arterioles).

Additions to Section 3.4 are provided next:

*The impact of autoregulatory mechanisms on transmural flow distribution (ENDO/EPI) (**Figure 11B**) reveals that the myo only, meta only, and myo+meta cases exhibit behavior qualitatively consistent with the baseline case across perfusion pressures. Conversely, the ENDO/EPI ratio in the shear-only case exhibits a distinct behavior, characterized by high sensitivity to minor fluctuations in perfusion pressure around the homeostatic pressure. This suggests that shear-dependent mechanisms alone are insufficient for regulating transmural flow distribution.*

*Mild ($p^{in} = 59$ mmHg, **Figure 11C**) and severe ($p^{in} = 38$ mmHg, **Figure 11D**) reductions in perfusion pressures produce changes in subepi vessel diameters (ΔD) that are qualitatively similar to each other for all combinations of autoregulatory mechanisms considered. For $D_h > 160$ µm, the myo only and myo+meta cases closely follow the baseline curve, whereas the meta only and shear only cases experience greater constriction. This is indicative of myogenic control being the most influential in determining the vascular response for vessels $D_h > 160$ µm. For vessels $50 < D_h < 160$ µm, the myo+meta case experiences the greatest dilation, due to the absence of the constrictive stimulus provided by the shear-dependent mechanism. Conversely, the shear only case demonstrates the most pronounced constriction, as the reduction in perfusion pressure leads to a decrease in flow (**Figure 11A**) and a consequent loss of shear-mediated dilatory stimulus. The myo only case best follows the baseline curve in the $50 < D_h < 160$ µm range, also indicative of the myogenic response being the dominant mechanism in this diameter range. However, for $D_h < 50$ µm, the myo only case becomes more constricted than the baseline and converges toward the shear only curve. By contrast, both the meta only and myo+meta cases both converge with the baseline case in this diameter range. This behavior indicates that the metabolic mechanism is the dominant regulator for the smallest vessels, with myogenic and shear-dependent mechanisms playing a diminished role.*

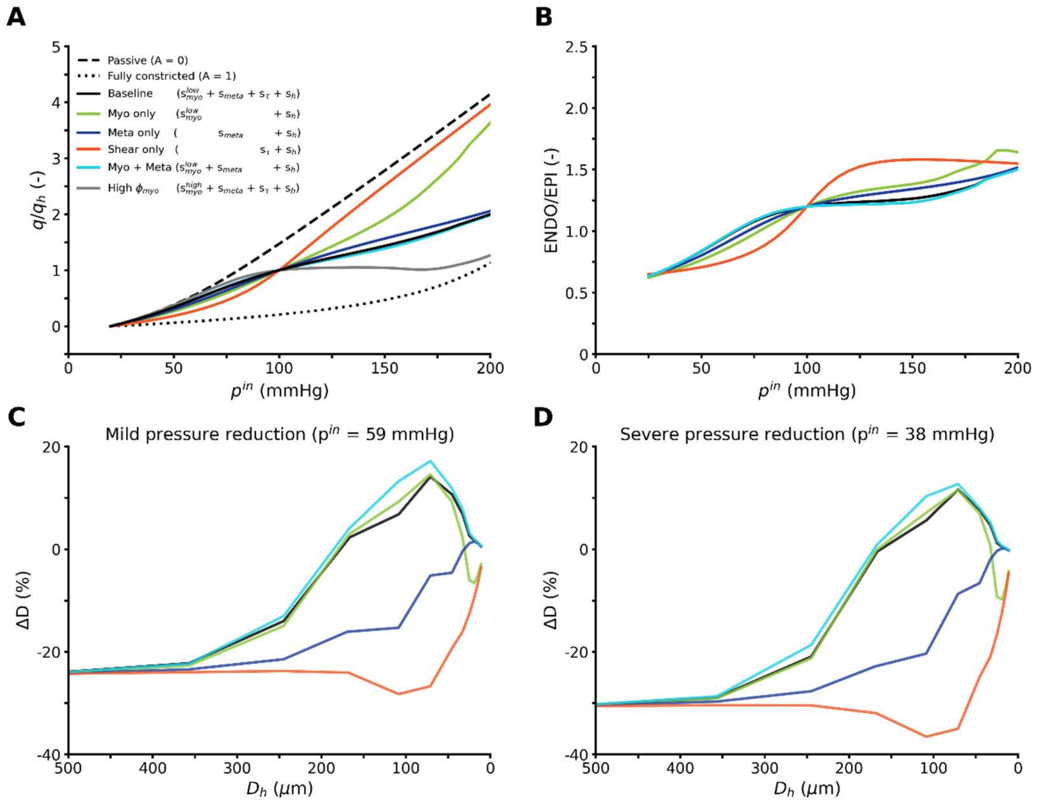

Updated figure 11. Impact of autoregulatory mechanisms on (**A**) the coronary pressure-flow relationship, (**B**) transmural flow distribution (ENDO/EPI), and subepi vascular diameter responses to (**C**) mild and (**D**) severe reductions in perfusion pressure.

Additions to Section 3.5 are provided next:

> *The impact of these mechanical changes on transmural flow distribution (**Figure 12B**) shows that collagen stiffening produces a flattening of the ENDO/EPI ratio curve following deviations in p^{in} when compared to the baseline. SMC dysfunction causes the ENDO/EPI ratio to increase for $p^{in} > 100$ mmHg, but shows little change for $p^{in} < 100$ mmHg. Both collagen stiffening and SMC dysfunction reduce the magnitude of ΔD in response to mild (**Figure 12C**) and severe (**Figure 12D**) reductions in perfusion pressure. This demonstrates that both vascular dysfunctions limit the ability of the vessel to modulate diameter, as evidenced by the narrowing between the passive and fully constricted curves in **Figure 12A**. Note, changing the passive and active material properties influences the homeostatic morphology of the tree, as evident by the different homeostatic diameters in **Figure 12C** and **Figure 12D**.*

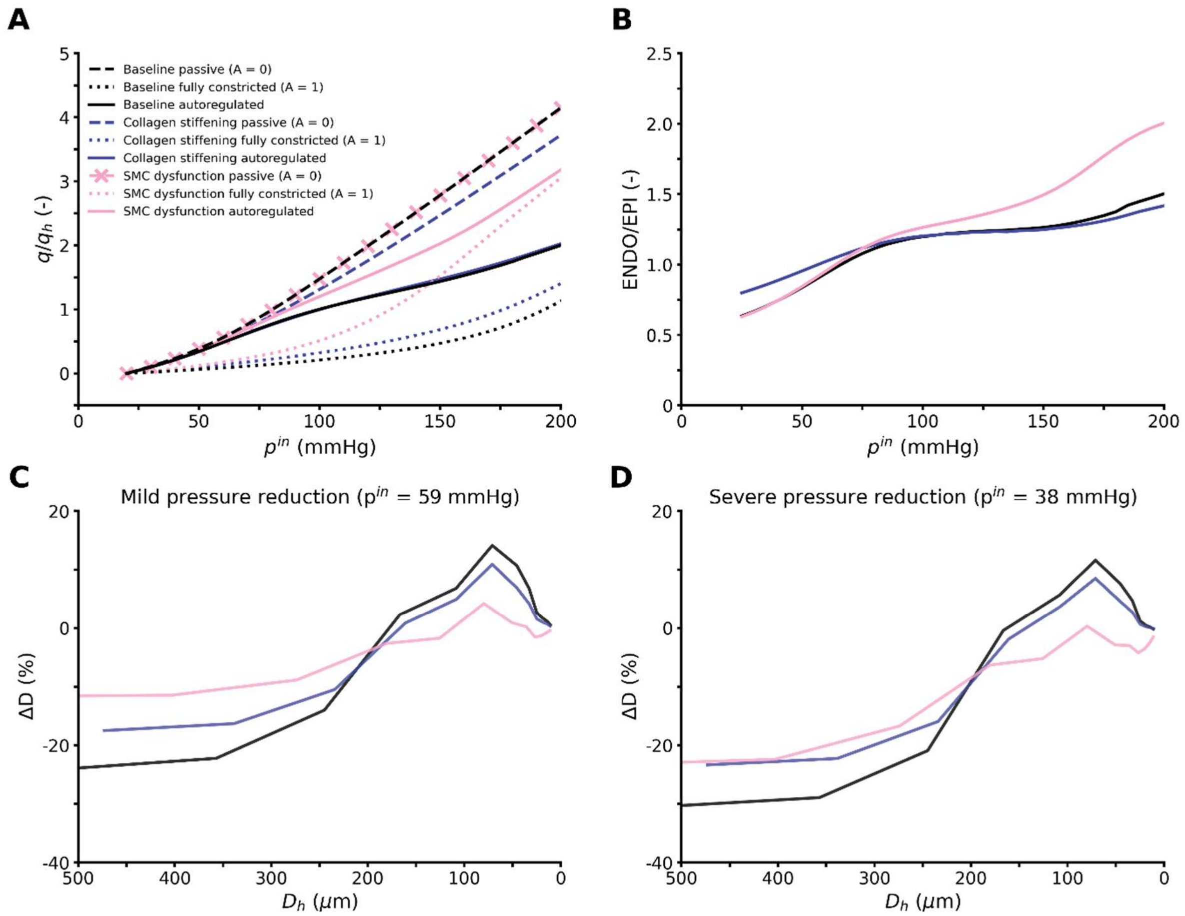

Updated figure 1: Analysis on how changes in vascular mechanical properties impact (A) coronary pressure-flow relationships, (B) transmural flow distribution (ENDO/EPI), and vascular diameter responses to (C) mild and (D) severe reductions in perfusion pressure.

Updated table 1: Constrained mixture model material parameters that are constant across vessel sizes.

Parameter	Description	Unit	Value	X_1 Sensitivity (%)			X_2 Sensitivity (%)			X_3 Sensitivity (%)	Source
				Subepi	Midwall	Subendo	Subepi	Midwall	Subendo		
c_1	Elastin material parameter	Pa/kg	94.5	1.0	0.8	0.4	<0.1	<0.1	<0.1	1.4	Calibrated (Stage 1)
c_2	Collagen material parameter	Pa/kg	308.6	24.2	24.9	11.3	0.4	0.4	0.3	0.2	Calibrated (Stage 1)
c_3	Collagen dimensionless material parameter	-	3.9	9.5	21.6	16.5	0.1	0.1	<0.1	0.6	Calibrated (Stage 1)
c_4	SMCs material parameter	Pa/kg	57.2	12.4	13.9	8.2	0.1	0.1	0.3	0.3	Calibrated (Stage 1)
c_5	SMCs dimensionless material parameter	-	0.59	<0.1	0.4	0.3	<0.1	<0.1	<0.1	0.7	Calibrated (Stage 1)
S_{max}	SMC maximum stress	kPa	250	90.8	135.5	134.1	4.0	4.0	4.3	9.5	(Rachev & Hayashi, 1999; Baek *et al.*, 2007)
λ_0	Zero active tension stretch	-	0.4	15.8	22.8	15.6	<0.1	<0.1	<0.1	0.1	(Rachev & Hayashi, 1999; Baek *et al.*, 2007)
λ_M	Maximum active tension stretch	-	1.1	87.2	123.7	107.0	0.8	0.8	0.8	3.2	(Rachev & Hayashi, 1999; Baek *et al.*, 2007)
ρ_{wall}	Vessel density	kg/m^3	1060	-	-	-	-	-	-	-	-
L_0	Metabolic control characteristic decay length	m	0.001	-	-	-	-	-	-	-	(Namani *et al.*, 2018)

Updated table 2 (previously in table 1): Constrained mixture model autoregulatory parameters that are constant across vessel sizes.

Parameter	Description	Unit	Value	X_1 Sensitivity (%)	X_2 Sensitivity (%)	X_3 Sensitivity (%)	Source
Subepi							
a_{myo}	Myogenic scaling coefficient	-	6.32	101.4	-	<0.1	Calibrated (Stage 1)
a_{meta}	Metabolic scaling coefficient	-	2.82	-	-	0.9	Calibrated (Stage 3)
a_τ	Shear-dependent scaling coefficient	-	1.58	-	-	0.7	Calibrated (Stage 3)
Midwall							
a_{myo}	Myogenic scaling coefficient	-	5.43	99.6	-	0.7	Calibrated (Stage 1)
a_{meta}	Metabolic scaling coefficient	-	2.82	-	-	0.9	Calibrated (Stage 3)
a_τ	Shear-dependent scaling coefficient	-	1.58	-	-	0.7	Calibrated (Stage 3)
Subendo							
a_{myo}	Myogenic scaling coefficient	-	4.37	50.8	-	<0.1	Calibrated (Stage 1)
a_{meta}	Metabolic scaling coefficient	-	2.82	-	-	0.9	Calibrated (Stage 3)
a_τ	Shear-dependent scaling coefficient	-	1.58	-	-	0.7	Calibrated (Stage 3)

Updated table 3 (previous table 2): Constrained mixture model parameters that are variable across vessel sizes. Small artery: SA, large arteriole: Lao, intermediate arteriole: IAo, and small arteriole: SAo.

Parameter	Description	Unit	Value				X_1 Sensitivity (%)				X_2 Sensitivity (%)				X_3 Sensitivity (%)			
			SA	LAo	IAo	SAo	SA	LAo	IAo	SAo	SA	LAo	IAo	SAo	SA	LAo	IAo	SAo
																Subepi		
G_θ^e, G_z^e	Elastin pre-stretch	-	1.00	1.29	1.13	1.16	17.6	3.0	2.2	7.3	0.4	<0.1	0.1	0.2	0.2	<0.1	<0.1	2.5
G_h^c	Collagen pre-stretch	-	1.10	1.02	1.00	1.00	170.1	74.0	73.8	113.8	4.7	0.2	0.1	2.7	1.1	0.3	0.4	36.7
G_h^m	SMCs pre-stretch	-	1.00	1.00	1.00	1.00	11.6	38.8	51.9	20.1	1.5	0.1	0.1	0.6	0.2	0.2	0.3	7.7
υ^e	Elastin mass fraction	-	0.07	0.02	0.02	0.04	1.3	0.2	0.3	1.2	<0.1	<0.1	<0.1	<0.1	0.1	<0.1	<0.1	0.1
υ^c	Collagen mass fraction	-	0.36	0.32	0.30	0.59	17.4	18.4	6.8	78.6	0.4	<0.1	<0.1	<0.1	0.2	0.1	<0.1	2.6
υ^{m*}	SMCs mass fraction	-	0.57	0.66	0.68	0.37	-	-	-	-	-	-	-	-	-	-	-	-
ϕ_{myo}	Normalized myogenic strength	-	0.70	0.60	1.00	0.86	-	-	-	-	-	-	-	-	-	-	-	-
ϕ_τ	Normalized myogenic strength	-	0.62	1.0	0.83	0.42	-	-	-	-	-	-	-	-	-	-	-	-
																Midwall		
G_θ^e, G_z^e	Elastin pre-stretch	-	1.01	1.33	1.12	1.07	14.6	2.5	1.7	17.0	0.3	<0.1	0.1	0.3	0.9	0.7	0.7	3.8
G_h^c	Collagen pre-stretch	-	1.10	1.04	1.00	1.00	168.3	106.5	93.2	132.5	4.6	0.2	<0.1	2.3	1.9	1.0	1.0	32.4
G_h^m	SMCs pre-stretch	-	1.00	1.02	1.00	1.00	20.6	44.7	62.6	31.1	1.7	0.1	0.1	0.6	0.9	0.9	1.0	8.7
υ^e	Elastin mass fraction	-	0.05	0.02	0.02	0.05	1.1	<0.1	0.2	3.5	<0.1	<0.1	<0.1	<0.1	0.6	0.7	0.7	0.6
υ^c	Collagen mass fraction	-	0.34	0.30	0.28	0.54	26.5	20.3	8.5	77.1	0.4	<0.1	<0.1	<0.1	0.5	0.6	0.6	1.6
υ^{m*}	SMCs mass fraction	-	0.61	0.67	0.70	0.41	-	-	-	-					-	-	-	-
ϕ_{myo}	Normalized myogenic strength	-	0.84	0.70	1.00	0.75	-	-	-	-					-	-	-	-
ϕ_τ	Normalized myogenic strength	-	0.62	1.0	0.83	0.42	-	-	-	-					-	-	-	-
																Subendo		
G_θ^e, G_z^e	Elastin pre-stretch	-	1.00	1.37	1.20	1.12	22.2	2.4	1.7	10.9	0.5	<0.1	<0.1	0.2	0.3	<0.1	<0.1	2.2
G_h^c	Collagen pre-stretch	-	1.10	1.03	1.00	1.00	72.9	79.5	81.6	98.2	3.4	0.2	0.1	1.9	0.6	0.2	0.3	24.9
G_h^m	SMCs pre-stretch	-	1.07	1.03	1.00	1.00	32.4	44.1	57.4	31.3	2.8	0.2	0.1	0.7	0.4	0.2	0.3	7.5
υ^e	Elastin mass fraction	-	0.09	0.02	0.02	0.04	2.4	0.3	0.2	0.9	<0.1	<0.1	<0.1	<0.1	0.1	<0.1	<0.1	0.1
υ^c	Collagen mass fraction	-	0.19	0.23	0.22	0.48	7.7	11.0	5.5	52.3	0.3	<0.1	<0.1	<0.1	0.1	<0.1	<0.1	1.1
υ^{m*}	SMCs mass fraction	-	0.72	0.75	0.76	0.48	-	-	-	-					-	-	-	-
ϕ_{myo}	Normalized myogenic strength	-	0.92	0.74	1.00	0.66	-	-	-	-					-	-	-	-
ϕ_τ	Normalized myogenic strength	-	0.62	1.0	0.83	0.42	-	-	-	-					-	-	-	-

$*\upsilon^m = 1 - \upsilon^e - \upsilon^c$

Comment 6: The framework is positioned for pathophysiological studies, yet no disease case is demonstrated. Including at least one illustrative pathological scenario (e.g., impaired myogenic tone, collagen stiffening, endothelial dysfunction) would enhance the impact.

Response: We respectfully disagree with the reviewer in that no disease case was demonstrated in our manuscript. The following pathophysiological scenarios were included:

1. Collagen stiffening (Section 3.5). We investigated the impact of collagen stiffening by doubling the collagen material parameter c_2. We found that stiffening of the collagen fibers results in upward and downward shifts of the fully constricted and dilated flow-pressure curves, respectively. However, coronary autoregulation remains relatively intact, suggesting that collagen stiffening constrains the dilatory capacity of the coronary tree without abolishing its autoregulatory function.
2. SMC dysfunction (Section 3.5). We modeled the impact of SMC dysfunction by halving the maximum SMC stress parameter S_{max}. Impaired SMC contractility produced a loss of coronary autoregulation accompanied by an upward shift in the fully constricted flow-pressure curve (**Figure 12A**). This indicates that reduced SMC contractile capacity severely compromises the ability of the coronary circulation to autoregulate.
3. Impaired autoregulatory mechanisms (Section 3.4). The impact of impaired autoregulatory mechanisms was also explored. We considered six different cases in **Figure 11**, labeled baseline, myo only, meta only, shear only, myo+meta, and high ϕ_myo. From our analysis, we concluded that the relative contributions of the autoregulatory mechanisms follow the order: metabolic > myogenic > shear-dependent. These results suggest that impaired metabolic control yields greater autoregulatory consequences than impaired shear-dependent control.

As the reviewer indicates, this framework is well suited to study pathophysiological responses. Ultimately, the examples above would have to be informed by experimental data. Section 4.5 has been updated to better frame the analyses above as a demonstration of the framework's pathophysiological modeling potential.

Minors:

Comment 1: There are several image-based computational studies on coronary fluid-solid interaction and network biomechanics. Including a concise review of these works in the Introduction or Discussion would better contextualize the contribution.

Response: We agree with the reviewer that situating our work relative to prior image-based computational studies on coronary fluid-solid interaction and network biomechanics strengthens the context for our contribution. Accordingly, we have added to the beginning of the Discussion section to provide background on image-based studies of epicardial coronary hemodynamics, including fluid-structure interaction and network hemodynamics studies.

Comment 2: If "wall stress" refers to wall shear stress, terminology should be unified throughout the manuscript to avoid confusion with circumferential or hoop stress.

Response: We appreciate the reviewer for catching this point of confusion. We intended to use the term 'hoop stress' throughout the manuscript to refer to the circumferential (tensile) stress in the wall. However, as the reviewer indicates, there were two instances in the manuscript where we used "wall stress", instead of the intended "hoop stress", which avoids any potential confusion with "wall shear stress". We fixed this problem in the updated manuscript, using the 'hoop stress' terminology throughout.

Comment 3: "In additional to" should be corrected to "In addition to".

Response: Thank you. We have corrected this error in the manuscript.

Comment 4: A brief rationale for selecting 12 generations would help readers understand anatomical completeness.

Response: We agree with the reviewer that providing a rationale for selecting the 12 generations would help readers understand anatomical completeness.

We used 12-generation trees because when prescribing literature-based terminal flow rates of 0.001375, 0.0015, and 0.00165 mm^3/s for the subepicardial, midwall, and subendocardial layers, respectively, the 12-generation configuration provided the best overall agreement with reported flow–diameter and wall shear stress–diameter distributions (see Figure 6). To clarify this modeling decision, we have added a brief rationale for selecting 12 generations in Section 2.6 (Framework calibration).

Comment 5: A clear ranking or sensitivity-based decomposition of passive element contributions (e.g., elastin vs collagen vs passive SMC) would help interpret whether predicted vessel behavior aligns with known biomechanical literature.

Response: We agree with the reviewer that providing a sensitivity-based decomposition of passive element contributions would help interpret whether predicted vessel behavior aligns with known biomechanical literature.

Utilizing the pressure-diameter sensitivity metric X_1 (described in our answer to Major Comment 5), the sensitivity of passive material properties is $c_2 > c_3 > c_4 > c_1 > c_5$, when averaged across the three trees. The high sensitivity to collagen (parameters c_2 and c_3) aligns with collagen's highly non-linear behavior and its substantial contribution to the vessel wall mass (Figure 5e) (Humphrey, 2002). The passive SMC parameter c_4 is the third most sensitive, while the SMC non-linearity parameter c_5 is the least sensitive. Although SMCs contribute minimally to the passive mechanics of large elastic arteries (Lin *et al.*, 2022), they are believed to play a more prominent structural role in muscular vessels (Leloup *et al.*, 2015). Our Stage 1 calibration identifies a low elastin content, supporting that elastin plays a lesser role in the passive mechanics of the coronary microvasculature. This is reflected by c_1 being the second least sensitive passive parameter.

We have updated Section 4.1 (Constrained mixture model parameter estimation) to discuss how our calibrated model aligns with the biomechanical literature using the X_1 sensitivity metric.

Referee #2
The manuscript by Eden et al. presents - to the best of my knowledge - a novel computational framework for coronary pressure-flow autoregulation based on constrained mixture models. A three-stage calibration uses swine myography data for passive and myogenic vessel wall properties, Murray's law optimization for homeostatic morphometry, and swine pressure-flow data for metabolic/shear-dependent scaling. An extended Womersley solution accounts for pulsatile hemodynamics under time-varying intramyocardial pressure. This is a very comprehensive contribution that is novel and interesting to the readers of J Phys.

Regarding impact and originality, to the best of my knowledge this is the first CMT-based coronary autoregulation framework, and its multi-scale capability is an advance over existing models. The model provides mechanistic insight into transmural autoregulatory heterogeneity and the dominance of metabolic control.

Regarding study design and validity, calibration and validation datasets are largely independent, though all calibration data derive from swine while comparisons are mostly to canine data, as mentioned below in the specific concerns. All data assessed in this manuscript are retrospective/previously published. I have no ethical concerns regarding this submission.

Overall, I recommend a revision addressing the following (minor) issues:

Response: The authors thank Referee #2 and appreciate their careful reading of the manuscript and helpful comments. We have carefully considered each point raised and have revised the manuscript accordingly, as detailed below.

Comment 1: Inter-species calibration while comparisons (e.g. Fig 10) were mostly made to canine data. There is limited data available for calibration and validation, but the authors should acknowledge this in the limitations section.

In that regard also note that in l801: "[...] wall shear stress tends to be greater than that measured by Stepp et al. (1999) in canines [32], but agrees better with previous modeling work by Cornelissen et al. (2002) [11]." Cornelissen et al. appears to be based on porcine data, which should be noted when drawing this comparison.

Response: The reviewer is correct that given the scarcity of literature data, inter-species data was used for calibration while canine data was primarily used for comparisons. We agree that this cross-species approach is a limitation of our calibration and validation process, given the inherent species variability in coronary physiology (Tune *et al.*, 2020). We have added this limitation to Section 4.6 (Model limitations).

The reviewer correctly identifies that the wall shear stress measurements reported by Stepp *et al.*, (1999) are in canine, while the shear stress values reported by Cornelissen *et al.*, (2002) are derived from a mathematical model based on porcine data. We agree that it is important to make this distinction when drawing comparison between these data sources. We have clarified in Section 3.1.2 that the wall shear stress measurements reported by Stepp *et al.*, (1999) are from canine measurements, and the modeling work by Cornelissen *et al.*, (2002) is based on porcine

data. We also acknowledge this important distinction in Section 4.2 of the Discussion.

Comment 2: Sensitivity analysis seems to be only assessed for stage 3? What about stage 1 and 2? Also the diameter exponent \xi seems to be a bit above the values reported by Kaimovitz et al. 2008. What would be the impact of this on downstream hemodynamics?

Response: We agree with the reviewer that providing a broader sensitivity analysis would strengthen the manuscript. This point was also raised by Reviewer #1, and our detailed response is provided above.

Briefly, we have added two new sensitivity metrics into all Tables (X_1 and X_2 sensitivity), to quantify the impact of model parameters on Stages 1 and 2 of calibration. Note, the previous Table 1 was split into two tables (now Table 1 and 2) to make room for the additional sensitivity data.

Of the three calibration stages, Stage 1 was generally the most sensitive to changes in passive and active material parameters, whereas the homeostatic coronary trees calibrated in Stage 2 were relatively insensitive to these perturbations (**Tables 1–3**). This discrepancy in sensitivity is likely because Stage 1 is evaluated over a wide range of perfusion pressures, which captures the nonlinear mechanical response of the vessel wall. In contrast, the Stage 2 objective function is evaluated at a single homeostatic perfusion pressure, where the material behavior is more constrained.

We have additionally expanded the sensitivity analysis presented in Figure 11 (impact of autoregulatory mechanisms) and Figure 12 (impact of vascular mechanical properties) to include the effects on transmural flow ratios and diameter changes under both mild and severe reductions in perfusion pressure. Text was added to Section 3.4 and Section 3.5 to discuss these results.

The reviewer correctly identifies a discrepancy between our calibrated values of the diameter exponent (ξ, Figure 6B) and that reported by in Kaimovitz *et al.*, (2008). We interpret this outcome as competing objectives within the homeostatic cost function (Equation D6 in Appendix D):

$$\boldsymbol{C_{total} = \sum_j C_j L_j N_j + k(\xi_{model} - \xi_{data})^2}, \quad \textbf{(D6)}$$

where the first term represents an extended Murray's law and the second term is the incorporation of diameter exponent measurement by Kaimovitz *et al.*, (2008). In isolation, the extended Murray's law yields an exponent of ~3 Gharahi *et al.*, (2023). The inclusion of the Kaimovitz *et al.*, (2008) dataset pulls the diameter exponent towards the experimental values, and our calibrated homeostatic diameter exponent represents a balance between the energy-based Murray's law and the experimental data.

Mathematically, a greater value of ξ means the reduction in diameter from the mother to daughter vessels is less pronounced, resulting in a lower vascular resistance in the daughter vessels. Therefore, our higher values of ξ relative to Kaimovitz *et al.*, (2008) suggest our model may exhibit lower resistance in the larger vessels, particularly for D_h > 65 μm. However, because

the model reproduces pressure-diameter (Figure 6G) and flow-diameter (Figure 6H) distributions in fair agreement with Kassab *et al.*, (1997), we believe our homeostatic optimization procedure produces a physiologically reasonable distribution of resistance across trees.

We have added to Section 4.2 (Morphometry, structure, and hemodynamics of homeostatic coronary trees) to add to our discussion on the differences in our diameter exponent between our model and Kaimovitz *et al.*, (2008).

Comment 3: Additional papers worth looking at could be: 1. Namani et al. 2020, DOI: 10.1152/ajpheart.00563.2019, overview of coronary autoregulation modeling; 2. Hoffman & Spaan (1990), DOI: 10.1152/physrev.1990.70.2.331, potentially in Section 4.4; or Wu et al. (2021), DOI: 10.1016/j.jmbbm.2020.104161, for determining constituent pre-stretches in CMT vascular models.

Response: We thank the reviewer for providing additional papers which strengthen the manuscript and provide valuable context for how our work fits into broader literature. We now reference Namani *et al.*, (2020) in Section 4, Hoffman & Spaan, (1990) in Section 4.4, and Wu *et al.*, (2021) in Section 4.2.

REFERENCES

Baek S, Valentín A & Humphrey JD (2007). Biochemomechanics of Cerebral Vasospasm and its Resolution: II. Constitutive Relations and Model Simulations. *Ann Biomed Eng* **35,** 1498–1509.

Boatwright RB, Downey HF, Bashour FA & Crystal GJ (1980). Transmural variation in autoregulation of coronary blood flow in hyperperfused canine myocardium. *Circ Res* **47,** 599–609.

Canty JM (1988). Coronary pressure-function and steady-state pressure-flow relations during autoregulation in the unanesthetized dog. *Circ Res* **63,** 821–836.

Cornelissen AJM, Dankelman J, VanBavel E & Spaan JAE (2002). Balance between myogenic, flow-dependent, and metabolic flow control in coronary arterial tree: a model study. *Am J Physiol-Heart Circ Physiol* **282,** H2224–H2237.

Dick GM, Namani R, Patel B & Kassab GS (2018). Role of Coronary Myogenic Response in Pressure-Flow Autoregulation in Swine: A Meta-Analysis With Coronary Flow Modeling. *Front Physiol* **9,** 580.

Gharahi H, Filonova V, Mullagura HN, Nama N, Baek S & Figueroa CA (2023). A multiscale framework for defining homeostasis in distal vascular trees: applications to the pulmonary circulation. *Biomech Model Mechanobiol* **22,** 971–986.

Guo X & Kassab GS (2004). Distribution of stress and strain along the porcine aorta and coronary arterial tree. *Am J Physiol-Heart Circ Physiol* **286,** H2361–H2368.

Hoffman JI & Spaan JA (1990). Pressure-flow relations in coronary circulation. *Physiol Rev* **70,** 331–390.

Humphrey JD (2002). *Cardiovascular Solid Mechanics: Cells, Tissues, and Organs*. Springer, New York, NY. Available at: https://link.springer.com/10.1007/978-0-387-21576-1 [Accessed March 27, 2025].

Kaimovitz B, Huo Y, Lanir Y & Kassab GS (2008). Diameter asymmetry of porcine coronary arterial trees: structural and functional implications. *Am J Physiol-Heart Circ Physiol* **294,** H714–H723.

Kanatsuka H, Lamping KG, Eastham CL & Marcus ML (1990). Heterogeneous changes in epimyocardial microvascular size during graded coronary stenosis. Evidence of the microvascular site for autoregulation. *Circ Res* **66,** 389–396.

Kassab GS, Berkley J & Fung Y-CB (1997). Analysis of pig's coronary arterial blood flow with detailed anatomical data. *Ann Biomed Eng* **25,** 204–217.

Leloup AJA, Van Hove CE, Heykers A, Schrijvers DM, De Meyer GRY & Fransen P (2015). Elastic and Muscular Arteries Differ in Structure, Basal NO Production and Voltage-Gated Ca2+-Channels. *Front Physiol*; DOI: 10.3389/fphys.2015.00375.

Liao JC & Kuo L (1997). Interaction between adenosine and flow-induced dilation in coronary microvascular network. *Am J Physiol-Heart Circ Physiol* **272,** H1571–H1581.

Lin C-J, Cocciolone AJ & Wagenseil JE (2022). Elastin, arterial mechanics, and stenosis. *Am J Physiol - Cell Physiol* **322,** C875–C886.

Namani R, Kassab GS & Lanir Y (2018). Integrative model of coronary flow in anatomically based vasculature under myogenic, shear, and metabolic regulation. *J Gen Physiol* **150,** 145–168.

Namani R, Lanir Y, Lee LC & Kassab GS (2020). Overview of mathematical modeling of myocardial blood flow regulation. *Am J Physiol-Heart Circ Physiol* **318,** H966–H975.

Pries AR, Secomb TW, Gessner T, Sperandio MB, Gross JF & Gaehtgens P (1994). Resistance to blood flow in microvessels in vivo. *Circ Res* **75,** 904–915.

Rachev A & Hayashi K (1999). Theoretical study of the effects of vascular smooth muscle contraction on strain and stress distributions in arteries. *Ann Biomed Eng* **27,** 459–468.

Stepp DW, Nishikawa Y & Chilian WM (1999). Regulation of Shear Stress in the Canine Coronary Microcirculation. *Circulation* **100,** 1555–1561.

Szafron JM, Yang W, Feinstein JA, Rabinovitch M & Marsden AL (2023). A computational growth and remodeling framework for adaptive and maladaptive pulmonary arterial hemodynamics. *Biomech Model Mechanobiol* **22,** 1935–1951.

Tune JD, Goodwill AG, Kiel AM, Baker HE, Bender SB, Merkus D & Duncker DJ (2020). Disentangling the Gordian knot of local metabolic control of coronary blood flow. *Am J Physiol Heart Circ Physiol* **318,** H11–H24.

Wu J, Augustin CM & Shadden SC (2021). Reconstructing vascular homeostasis by growth-based prestretch and optimal fiber deposition. *J Mech Behav Biomed Mater* **114,** 104161.

Dear Dr Figueroa,

Re: JP-RP-2026-291222R1 "**A Microstructurally-Motivated Framework to Study Autoregulation in the Coronary Circulation**" by Matthew J Eden, Hamid Gharahi, Victoria E Sturgess, Domingo E Uceda, Greg Dick, Seungik Baek, Daniel A Beard, Johnathan D. Tune, and C. Alberto Figueroa

We are pleased to tell you that your paper has been accepted for publication in The Journal of Physiology.

The last Word (or similar) version of the manuscript provided will be used by the Production Editor to prepare your proof. When this is ready you will receive an email containing a link to Wiley's Online Proofing System. The proof should be thoroughly checked and corrected as promptly as possible.

Authors should note that it is too late at this point to offer corrections prior to proofreading. Major corrections at proof stage, such as changes to figures, will be referred to the Editors for approval before they can be incorporated. Only minor changes, such as to style and consistency, should be made at proof stage. Changes that need to be made after proof stage will usually require a formal correction notice.

All queries at proof stage should be sent to: TJP@wiley.com.

Following copyediting and typesetting the accepted version will be published online.

Yours sincerely,

Natalia Trayanova
Senior Editor
The Journal of Physiology

IMPORTANT POINTS TO NOTE FOLLOWING ACCEPTANCE OF YOUR PAPER:

• IMPORTANT NOTICE ABOUT OPEN ACCESS: To assist authors whose funding agencies mandate immediate public access to published research findings, The Journal of Physiology allows authors to pay an Open Access (OA) fee to have their papers made freely available immediately on publication.

The Corresponding Author will receive an email from Wiley with details on how to register or log in to Wiley Authors where you will be able to place an order.

• You can check if your funder or institution has a Wiley Open Access Account here:
https://authors.wiley.com/author-resources/Journal-Authors/open-access/author-compliance-tool.html

• TRANSPARENT PEER REVIEW POLICY: To improve the transparency of its peer review process, The Journal of Physiology publishes online as supporting information the peer review history of all articles accepted for publication. Readers will have access to decision letters, including Editors' comments and referee reports, for each version of the manuscript as well as any author responses to peer review comments. Referees can decide whether or not they wish to be named on the peer review history document.

• You can help your research get the attention it deserves! Check out Wiley's free Promotion Guide for best-practice recommendations for promoting your work at: www.wileyauthors.com/eeo/guide. You can learn more about Wiley Editing Services which offers professional video, design, and writing services to create shareable video abstracts, infographics, conference posters, lay summaries, and research news stories for your research at: www.wileyauthors.com/eeo/promotion.

• If you would like to receive our 'Research Roundup', a monthly newsletter highlighting the cutting-edge research published in The Physiological Society's family of journals (The Journal of Physiology, Experimental Physiology, Physiological Reports, The Journal of Nutritional Physiology and The Journal of Precision Medicine: Health and Disease), please click this link, fill in your name and email address and select 'Research Roundup':
https://www.physoc.org/journals-and-media/membernews

EDITOR COMMENTS

Reviewing Editor:

Both reviewers agreed that the revision has satisfactorily addressed all of their concerns. This work presents a constrained framework for coronary autoregulation, integrating vessel wall mechanics with most of relevant variables. The model reproduces key physiological features, including autoregulatory responses and flow distributions, and aprovides a unified framework linking short-term tone regulation with long-term vascular remodeling. The study offers a valuable platform for future research in coronary physiology.

REFEREE COMMENTS

Referee #1:

The authors have satisfactorily addressed all of the concerns I raised in my initial review. I have no further comments.

Referee #2:

The authors have now addressed all of my concerns raised in the initial review and have satisfactorily revised the manuscript accordingly.
